# The Largest Chinese Cohort Study Indicates Homologous Recombination Pathway Gene Mutations as Another Major Genetic Risk Factor for Colorectal Cancer with Heterogeneous Clinical Phenotypes

**DOI:** 10.34133/research.0249

**Published:** 2023-10-17

**Authors:** Yun Xu, Kai Liu, Cong Li, Minghan Li, Fangqi Liu, Xiaoyan Zhou, Menghong Sun, Megha Ranganathan, Liying Zhang, Sheng Wang, Xin Hu, Ye Xu

**Affiliations:** ^1^ Department of Colorectal Surgery, Fudan University Shanghai Cancer Center, Shanghai, P.R. China.; ^2^ Department of Pathology, Fudan University Shanghai Cancer Center, Shanghai, P.R. China.; ^3^ Department of Pathology, Tissue Bank, Fudan University Shanghai Cancer Center, Shanghai, P.R. China.; ^4^ Department of Medicine, Memorial Sloan Kettering Cancer Center, New York, NY, USA.; ^5^ Department of Pathology and Laboratory Medicine, David Geffen School of Medicine, University of California, Los Angeles, CA, USA.; ^6^ Precision Cancer Medical Center, Fudan University Shanghai Cancer Center, Shanghai, P.R. China.

## Abstract

While genetic factors were associated with over 30% of colorectal cancer (CRC) patients, mutations in CRC-susceptibility genes were identified in only 5% to 10% of these patients. Besides, previous studies on hereditary CRC were largely designed to analyze germline mutations in patients with single genetic high-risk factor, which limited understanding of the association between genotype and phenotypes. From January 2015 to December 2018, we retrospectively enrolled 2,181 patients from 8,270 consecutive CRC cases, covering 5 categories of genetic high-risk factors. Leukocyte genomic DNA was analyzed for germline mutations in cancer predisposition genes. The germline mutations under each category were detected and analyzed in association with CRC susceptibility, clinical phenotypes, and prognoses. A total of 462 pathogenic variants were detected in 19.3% of enrolled CRC patients. Mismatch repair gene mutation was identified in 9.1% of patients, most prevalent across all high-risk groups. Homologous recombination (HR) gene mutations were detected in 6.5% of cases, penetrated in early-onset and extra-colonic cancer risk groups. Mutations in HR genes, including *BARD1*, *RAD50,* and *ATM,* were found to increase CRC risk with odds ratios of 2.8-, 3.1-, and 3.1-fold, respectively. CRC patients with distinct germline mutations manifested heterogeneous phenotypes in clinicopathology and long-term prognoses. Thus, germline mutation screenings should be performed for CRC patients with any of those genetic risk factors. This study also reveals that HR gene mutations may be another major driver for increased CRC risk.

## Introduction

Colorectal cancer (CRC) is one of the most prevalent malignancies globally with an estimated 1.87 million new cases diagnosed annually, and was the second leading cause of cancer-related death in 2020 [1,2]. In China alone, there are over 400,000 new CRC cases every year, and the incidence rate shows a consistent increase, with males witnessing a 2.5% annual rise and females at 1.5% [2,3]. Established general risk factors for CRC include age, sex, inflammatory bowel disease, and genetic variants susceptible to CRC [2]. Genetic influences account for over 30% of CRC incidences [4], but due to insufficient genetic screening, the germline mutations in high-penetrance CRC-susceptibility genes were found to account for only 5% to 10% of all CRCs [5,6]. Consequently, a vast majority of genes potentially predisposing individuals to CRC remain undiscovered.

Almost germline mutations linked to CRC susceptibility are identified in patients who exhibit specific genetic risk factors, which has been endorsed as indications for genetic screening, including early-onset CRC, family history of cancer, and deficient mismatch repair (dMMR) in tumor tissues by immunohistochemical (IHC) staining [7–11]. While tumors demonstrating dMMR through IHC suggest a strong likelihood of Lynch syndrome (LS) [12], both a family history of cancer [13] and early-onset cancer [8,10,14] serve as indicators of inherited cancer susceptibility. The National Comprehensive Cancer Network (NCCN) established management guidelines for genetic screening based on genetic analyses in CRC patients displaying these predominant genetic risk factors. However, these NCCN guidelines currently overlook approximately 28% of pathogenic and likely pathogenic (P/LP) variants [15]. Other substantial genetic risk factors associated with hereditary CRC, such as multiple primary CRC and primary hereditary cancer syndrome linked to extra-colonic cancer, were rarely studied [5,16]. Multiple primary cancers, encompassing both synchronous and metachronous CRC, along with extra-colonic cancers such as endometrial, ovarian, and pancreatic cancers, are frequently observed in LS and other hereditary cancer syndromes. However, due to a lack of robust supporting data, the current NCCN guidelines do not specifically address these 2 genetic risk factors. As a result, it is necessary to incorporate these genetic risk factors into the exploration of CRC-susceptibility genes.

Although many studies were reported on germline mutations for CRC patients with genetic risk factors, those works mostly evaluated a patient group(s) focusing on one single genetic risk factor. There is a lack of global views on genetic abnormalities that underlie the clinical high-risk CRC, and there is limited understanding of the association between germline mutations and the clinical genetic risk factors, and how the germline mutations contribute to the clinical characteristics and long-term outcomes for CRC patients. To address these important questions, we designed the study to investigate the germline mutations underlying the 5 clinical genetic risk factors: early-onset CRC, family history of cancer, dMMR of tumor tissues by IHC staining, multiple primary CRC, and primary hereditary cancer syndrome associated with extra-colonic cancer. To the best of our knowledge, this represents the first comprehensive study encompassing all 5 clinical genetic risk factors in CRC patients.

By admitting consecutive 8,270 cases of CRC patients at Fudan University Shanghai Cancer Center (FUSCC) over 4-year period from 2015 to 2018, we constituted the largest Chinese cohort for hereditary CRC study. Our aim was to develop a comprehensive understanding of genetic abnormalities in CRC patients with high-risk factors, and ascertain their contributions to clinical characteristics and outcomes for those individuals. Through an extensive germline mutation screening, we found that a significant proportion of patients with genetic risk factors had P/LP germline mutations. In addition to mutations in mismatch repair pathway (MMR) genes, remarkably a distinct group of P/LP germline mutations in homologous recombination (HR) pathway genes were also detected. Simultaneously, we uncovered that certain HR gene mutations contributed to an increased CRC risk. Furthermore, the expanded germline mutations identified in CRC patients were found to manifest heterogeneous phenotypes in clinicopathology, family cancer spectrum, cancer penetrance, and long-term prognoses. The findings of this study support the expansion of genetic screening for patients with the respective genetic risk factors, promoting early detection, prevention, and treatment for hereditary CRC.

## Results

### Clinical profiles of CRC patients with 5 distinct genetic risk factors

**Fig. 1.**
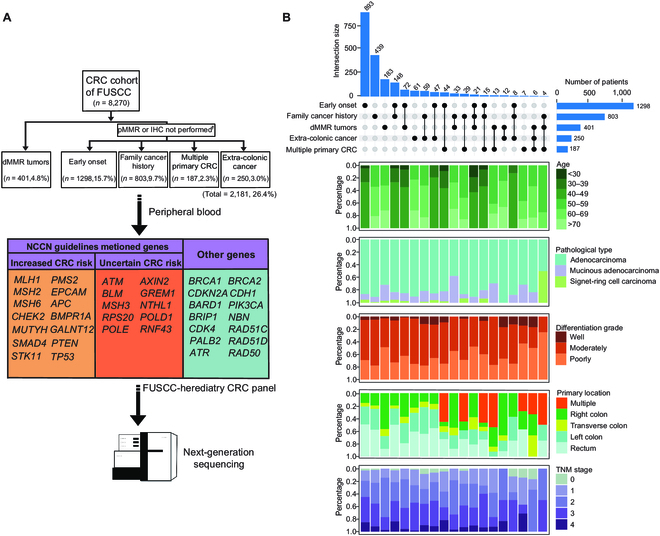
Schematic of the study and sample enrollment. (A) Schematic of the study. †Samples from patients with complete response after neoadjuvant therapy were not tested with IHC. Risk factors are not mutually exclusive. A patient fitting several criteria will be counted multiple times, causing the sum from all groups to surpass the total of 2,181 patients. (B) The upset plot illustrated enrolled samples' distribution and basic clinical characteristics.

To investigate germline mutations associated with all 5 genetic risk factors, we included 8,270 consecutive CRC patients in our study spanning 4 years from 2015 to 2018. In our consecutive CRC patient cohort, a substantial proportion exhibited genetic high-risk factors. Specifically, 26.4% (2,181 out of 8,270) of the CRC patients at FUSCC met our eligibility criteria. Patients with early-onset CRC made up 15.7% (1,296/8,270), followed by family history of cancer (9.7%,803/8,270), dMMR tumors (4.8%, 401/8,270), extra-colonic cancer (3.0%, 250/8,270), and multiple primary CRC (2.3%, 187/8,270) (Fig. 1A). Of the enrolled participants, 72.6% (1,583/2,181) had one genetic risk factor: early-onset CRC (40.9%, 893/2,181), family history of cancer (20.1%, 439/2,181), dMMR tumors (8.4%, 183/2,181), or extra-colonic cancer (2.8%, 61/2,181). Only 0.3% (7/2,181) of the patients had multiple primary CRC as their sole risk factor (Fig. 1B). Interestingly, a total of 27.4% (652/2,181) of the patients presented with at least 2 genetic risk factors. Patients across various genetic risk categories exhibited diverse clinicopathologic characteristics. For instance, those with multiple primary CRC frequently had an early onset or a family history of cancer. Patients with dMMR tumors often exhibited characteristics like mucinous adenocarcinoma, right-sided and poorly differentiated tumors, and a decreased percentage of TNM III/IV. Conversely, patients with early-onset or multiple primary CRC were more inclined to have a higher representation of TNM III/IV (Fig. 1B and Table S1).

**Table.** The definitions of genetic high-risk factor.

Genetic high-risk factor	Illustration
Early onset	Diagnosed before age of 50
dMMR	Tumor IHC manifesting dMMR
Multiple primary CRC	Synchronous and/or metachronous CRC at any age
Primary hereditary cancer syndrome associated extra-colonic cancer	Cancer associated with hereditary CRC:
	1. Extra-colonic cancer including upper gastrointestinal: gastric, small bowel, and gastro-esophageal junction
	2. Gynecologic: uterine and ovarian
	3. Urogenital: bladder, renal, and prostate
	4. Breast, hepatobiliary, and pancreatic
	5. Hematolymphoid, neurologic, and soft tissue
Family history of cancer	CRC and cancer associated with hereditary CRC susceptibility syndromes in first- and/or second-degree relatives at any age.

### Germline mutation prevalence in genetic high-risk CRC patients

To identify germline mutations in CRC patients with genetic risk factors, we utilized a hereditary cancer susceptibility gene panel consisting of 38 genes. Notably, P/LP variants were observed in 32 genes. The remaining 6 genes—*CDH1*, *CDK4*, *GREM1*, *GALNT12*, *RPS20*, and *BMPR1A*—exhibited no P/LP variants in our CRC cohort (Table S2). Among CRC patients presenting at least one of the 5 risk factors, 19.3% (421/2,181) had P/LP variants. Detailed examination revealed that 9.1% (199/2,181) harbored a P/LP variant within MMR pathway genes. Surprisingly, 4.2% (141/2,181) possessed a P/LP variant in HR pathway genes. Additionally, P/LP variants in the APC gene were detected in 0.73% (16/2,181) of the patients.

**Fig. 2.**
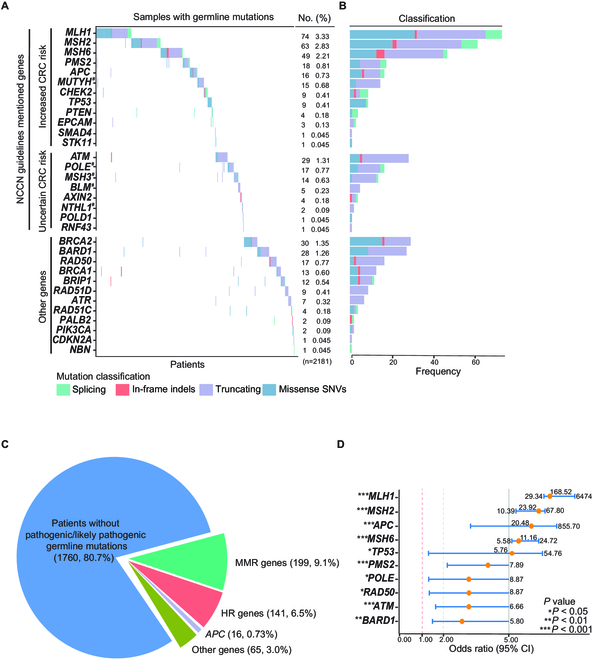
Germline mutation spectrum of 2,181 CRC patients with high genetic risk. (A) Landscape of P/LP germline mutations identified in CRC patients (*n* = 421), the 20 genes mentioned in NCCN guidelines are shown in the top part, and the other 12 genes are shown in the bottom part, #Heterozygous mutation. (B) According to mutation classification, count the frequency of mutations. (C) Overall detection rate of germline mutation and major gene pathways in high-risk patients (*n* = 2,181). (D) Forest plot displays the odds ratio of CRC susceptibility genes.

*MLH1* (3.33%, 74/2,181), *MSH2* (2.83%, 63/2,181), and *MSH6* (2.21%, 49/2,181) genes from the MMR pathway were the most frequently mutated genes in CRC patients. P/LP variants in CRC genes (NCCN guidelines) and “other genes” (not covered in the NCCN guidelines) were detected in 13.4% (335/2,181) and 5.8% (126/2,181) of our CRC patient cohort, respectively. P/LP from “other genes” that are not covered by the NCCN guidelines, contributed to more than 25% of all detected germline mutations (Fig. 2A). P/LP variants stemming from truncation or missense mutations represent the primary mutation types in most of these genes (Fig. 2B). P/LP variants from the moderate- and low-penetrance genes (labeled as “other genes” which are not covered in the NCCN guidelines) were detected in 3.0% of patients (65/2,181) (Fig. 2C).

To determine whether carrying P/LP variants in these genes confer an increased risk of CRC in the Chinese population, we performed a control-based risk analysis, which indicates that P/LP variants in the MMR pathway genes, *MLH1*, *MSH2*, *APC*, *MSH6*, *TP53*, and *PMS2*, were associated with an increased risk for CRC, consistent with what was known previously for LS [17–19]. Intriguingly, P/LP variants that belong to genes in the HR pathway, i.e., *RAD50*, *ATM,* and *BARD1*, were also found to be associated with an increased risk for CRC in our cohort. While P/LP variants in *MLH1*, *MSH2*, *APC*, *MSH6*, and *TP53* conferred a high risk for CRC with an odds ratio greater than 5, the P/LP variants in *PMS2*, *POLE*, *RAD50*, *ATM,* and *BARD1* were associated with a moderate risk increase with odds ratios greater than 2 but smaller than 5 (Fig. 2D and Table S3). These data reveal at the population level that patients with germline mutations in the HR pathway genes are susceptible to CRC, suggesting that mutations in HR pathway genes become another major contributor to increased risk of developing CRC.

### Germline mutations underlying different categories of genetic high-risk factors

**Fig. 3.**
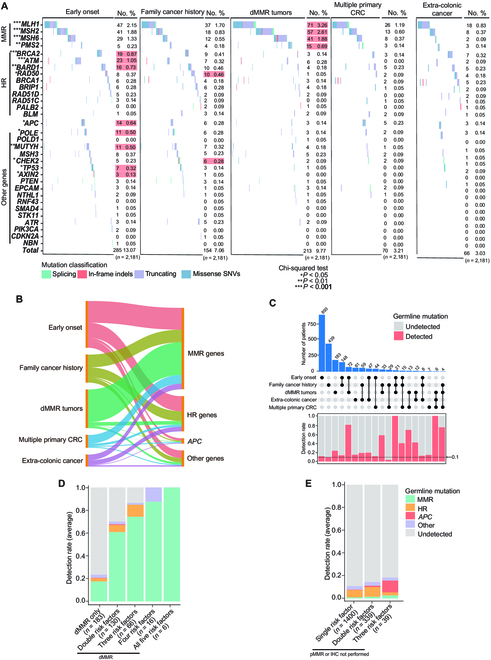
Germline mutation spectrum and prevalence with different genetic risks. (A) Landscape of P/LP germline mutation identified in patients with 5 genetic risk groups. The red background indicated the highest number of patients with the mutation. (B) Sankey plot illustrated the correlation between gene mutations and genetic risk factors. (C) Overall detection rate of germline mutation and major gene pathways in high-risk patients (*n* = 2,181). (D) The upset plot illustrated the enrolled sample’s distribution and germline mutation rate. (E) Relationship between dMMR tumors only or with at least one additional risk factor and the detection rate of germline mutation. The bar chart shows the average detection rate of germline mutation in patients with pMMR and other risk factors.

To understand the underlying germline mutations of distinct genetic high-risk factors in CRC patients, we performed an association analysis between these risk factors and the detection rates of P/LP variants in CRC or other cancer susceptibility genes. As expected, tumors exhibiting dMMR were most closely associated with the detection of P/LP variants in MMR genes. Early onset followed as the second leading risk factor for detecting mutations in the MMR genes: *MLH1*, *MSH2,* and *MSH6*. Both the presence of multiple primary CRCs and extra-colonic cancers similarly indicated a higher likelihood of identifying MMR gene mutations. Contrary to our expectations, HR pathway gene mutations predominated in groups with early onset, family cancer history, and extra-colonic cancers. P/LP variants in *APC*, *POLE*, *MUTYH*, *TP53,* and *AXIN2* were also significantly enriched in the early-onset risk group (Fig. 3A and B).

The frequency of germline mutations in CRC patients varied across different genetic high-risk factors. Examining each high-risk factor individually, patients exhibiting dMMR tumors displayed the most significant mutation detection rate at 23.5% (43/183). This was followed by the extra-colonic cancer group at 14.8% (9/61), the multiple primary CRC group at 14.3% (1/7), early-onset CRC at 11.4% (102/893), and the family cancer history group at 9.1% (40/439). Most risk groups exhibited a mutation detection rate surpassing 10% (Fig. 3C). Patients with dMMR tumors, combined with at least one other high-risk factor, exhibited a significantly higher likelihood of presenting germline mutations, with rates fluctuating between 40% and 100%. In patients with MMR proficient (pMMR) tumors, or those for whom IHC was not performed, the presence of additional risk factors indicated an escalating mutation detection rate, spanning from 10.8% to 18.4%. Notably, 3 pMMR CRC patients had 4 risk factors, yet none revealed detectable germline mutations (Fig. 3D and E). The mutation detection rates for patients exhibiting between 1 and 5 risk factors ranged from 12.3% to a striking 100% (Fig. S1).

### Clinical manifestations for different germline mutations

**Fig. 4.**
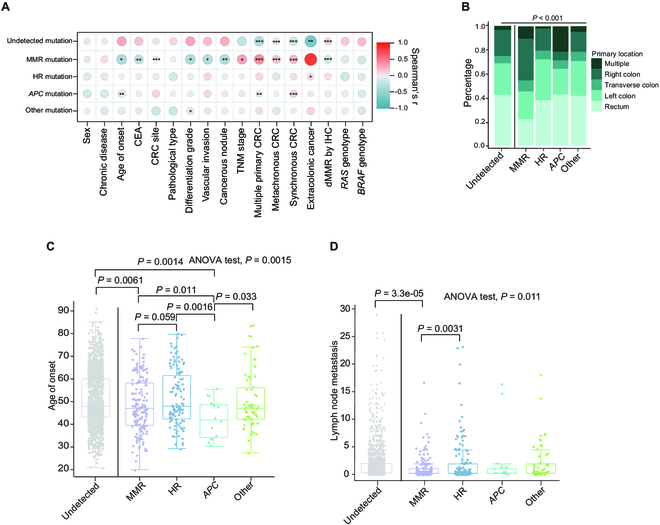
Clinical outcomes of patients carrying respective germline mutations. (A) Correlation analysis between mutation types and clinical outcomes. (B) Comparison of the proportion of CRC primary location in respective germline mutation carriers. (C) Age of onset distribution in individual germline mutation carriers. (D) Comparison of the number of metastasized lymph nodes observed in respective germline mutation carriers. MMR, HR, APC, and Other refer to LS patients, HR gene mutation carriers, FAP patients, and carriers of mutations in other genes, respectively. Adjusted *P* values were applied in multiple groups comparison.

To reveal the impact of germline mutations on clinical phenotype, we conducted an analysis correlating specific gene mutations with clinical features. Our results suggest that patients carrying different germline mutations manifested heterogeneous clinicopathologic characteristics. MMR gene mutations exhibited an inverse relationship with age, serum CEA levels, differentiation grade, vascular invasion, and TNM stage. Conversely, HR gene mutations were linked to the emergence of extra-colonic cancers, including breast, ovarian, urogenital, and gastric cancers (Fig. 4A and Fig. S2). Multiple primary CRC was predominantly identified in patients with familial adenomatous polyposis (FAP), and right-side colon cancer was most common in LS patients. Moreover, colorectal tumors in those harboring HR gene mutations tended to be located on the left side (Fig. 4B). The age of CRC onset in both LS and FAP patients was significantly earlier than in patients without any detected P/LP variants (Fig. 4C). LS patients diagnosed with CRC also had a significantly reduced number of metastatic lymph nodes in comparison to those with HR pathway gene mutations or those without any detected P/LP variants (with adjusted *P* values of *P* = 0.0031 and *P* = 3.3e−05, respectively). There was no statistically significant difference in the number of metastatic lymph nodes among CRC patients with other germline mutations (Fig. 4D and Tables S4 and S5).

### Prognoses of CRC patients with different germline mutations and risk factors

**Fig. 5.**
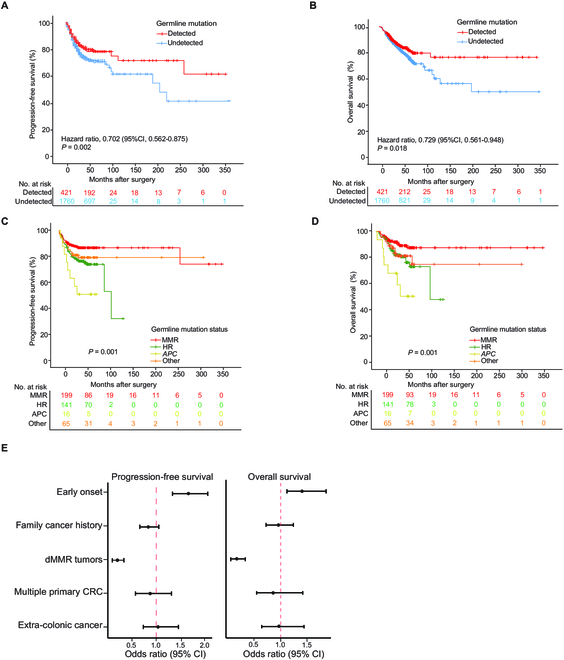
Survival analysis of respective molecular subtypes. (A) Overall survival curves for CRC patients carrying germline mutation. (B) Recurrence-free survival curves for CRC patients carrying germline mutation. (C) Overall survival curves for CRC of respective germline mutation status. (D) Recurrence-free survival curves for CRC of respective germline mutation status. (E) Risk ratio analysis of risk factors for survival. MMR, HR, APC, and Other refer to LS patients, HR genes mutation carriers, FAP patients, and carriers of mutations in other genes, respectively.

To understand the effects of germline mutations on long-term outcomes, we performed a survival analysis among patients exhibiting diverse gene mutations. In our cohort of CRC patients with a median follow-up duration of 53.7 ± 24.9 months, we investigated the relationship between germline mutations and long-term progression-free survival (PFS) and overall survival (OS). Among the CRC patients exhibiting at least one of the 5 study risk factors (*n* = 2,181), the 5-year PFS and OS rates stood at 71.0% and 79.8%, respectively. When focusing on CRC patients with germline mutations, their 5-year PFS and OS rates (77.1% and 83.1%, respectively) significantly surpassed those without mutations, which were 69.8% (χ^2^ = 9.976, *P* = 0.002) for PFS and 78.3% (χ^2^ = 5.591, *P* = 0.018) for OS (Fig. 5A and B). LS patients showcased a 5-year PFS rate of 84.8%, markedly outstripping HR gene mutation carriers (70.5%, χ^2^ = 9.971, *P* = 0.002) and FAP patients (50%, χ^2^ = 12.478, *P* < 0.001), but was comparable to CRC patients with other mutations (76.4%, χ^2^ = 2.045, *P* = 0.153). We observed a similar pattern in the 5-year OS. The 5-year OS of LS patients were 89.4%, which was higher than that of HR gene mutation carriers (81.2%, χ^2^ = 7.201, *P* = 0.007) and FAP patients (60.2%, χ^2^ = 16.676, *P* < 0.001), but was comparable to CRC patients with other mutations (81.7%, χ^2^ = 3.252, *P* = 0.071) (Fig. 5C and D).

The correlations between genetic risk factors and long-term OS and PFS rates indicated that the dMMR risk factor was associated with a better prognosis compared with other risk factors, and early age of onset predicted a poorer prognostic outcome (Fig. 5E and Table S6 and S7).

## Discussion

Although genetic testing is more accessible nowadays, the coverage of genetic screening for CRC patients remains insufficient. This has led to high proportion of germline mutation carriers that remain unidentified, which also hinders the implementation of precision treatment and cancer prevention [20,21]. This is the first study that enrolled high-risk hereditary CRC patients covering all categories of genetic high-risk factors for genetic testing. Through large-scale germline mutations screening, we discerned a notable prevalence of germline mutations in high-risk hereditary CRC patients. Each category of genetic high-risk factor is underlain by distinct germline mutations, with LS and HR gene mutations emerging as the most common. Notably, we first discovered some HR gene mutations contributing to increasing cancer risk. Furthermore, patients carrying different germline mutations manifested heterogeneous phenotypes in clinicopathology, family cancer spectrum, cancer penetrance, and long-term prognoses.

Our findings reveal that over 20% of CRC patients with at least one genetic risk factor are carriers of germline mutations. Previous studies have demonstrated that the prevalence of genes linked to CRC susceptibility in unselected patients ranges from 3% to 10% [6,22–24]. The selection criteria in our study, therefore, notably enhanced the detection efficacy for these mutation carriers. By incorporating rare genetic risk factors, specifically multiple primary CRC and extra-colonic cancer, we elevated the likelihood of identifying germline mutations by over twofold. Consequently, the presence of multiple primary CRC and extra-colonic cancer should be recognized as distinct criteria warranting genetic testing.

Looking into the detection rate for each category of genetic high-risk factors, we found that tumor IHC manifesting dMMR alone predicted a close to 20% probability of having LS while any one additional risk factor increases the probability of LS to more than 40%. Even though family cancer history is a key indicator of whether a CRC patient harbors germline mutations, the proportion of germline mutation carriers with family cancer history is similar to that of early onset. In clinical practice, family cancer history and significant phenotypes, such as several adenomatous or hamartomatous polyps, are indications for doctors to recommend genetic testing. However, other risk factors that may predict the presence of germline mutations have not been systematically studied. In this study, we found that MMR gene mutation was enriched in all risk groups, which indicated that these risk factors are significantly associated with LS. In addition, as other gene mutations were frequently detected, genetic risk factors including early-onset CRC, family cancer history, and extracolonic CRC may be associated with other hereditary cancer syndromes. These results indicate that mutations in some cancer susceptibility genes may lead to overlapping phenotypes of various hereditary cancer syndromes.

**Fig. 6.**
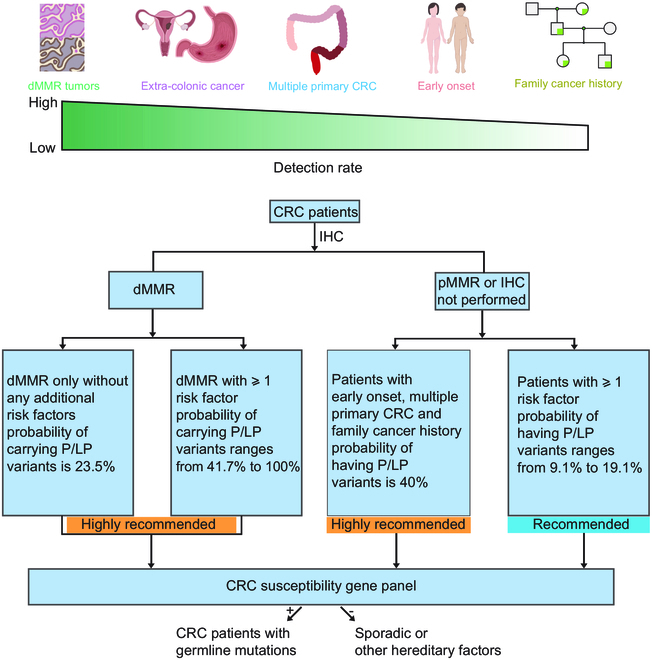
Establishment of genetic testing recommendations for patients carrying respective genetic risks. The above diagram illustrates the effect of 5 risk factors alone on predicting germline mutation carriers, and the flowchart shows genetic testing based on risk factors.

Our study also provides strong evidence for supplementing the guidelines for hereditary CRC genetic screening. In our cohort, more than 25% of all CRC patients had at least one of these 5 risk factors. dMMR tumors alone or in combination with one or more risk factors predicted a high probability (>20%) of harboring P/LP variants. We therefore highly recommend that patients with dMMR tumors have germline testing for CRC susceptibility genes, particularly the corresponding MMR genes. For patients with pMMR tumors or IHC not performed, early-onset CRC, family cancer history, and multiple primary CRC predicted a high probability (40%) of harboring P/LP variants. Therefore, germline testing is highly recommended for these groups of patients. CRC patients with extra-colonic cancer, early-onset CRC, family cancer history, or multiple primary CRC alone appeared to have a relatively low probability of carrying P/LP variants (<20%). However, genetic testing may still be recommended on an individualized basis depending on personal and family history (Fig. 6). Thus, genetic screening is recommended for patients carrying any one of the 5 categories of genetic risks.

In the current study, we offer a comprehensive view of the germline mutation landscape among CRC patients with genetic risk factors and assess the potential of these commonly mutated genes to elevate CRC risk. Our findings highlight that mutations in MMR genes, namely, the LS, are the most prevalent hereditary CRC in China. Intriguingly, HR gene mutations emerged as the second most frequently detected genetic abnormality in CRC patients. These findings not only expand our knowledge of germline mutations in hereditary CRC, but also lay a foundation for developing potentially targeted treatment strategies for CRC patients carrying HR gene mutations.

Mutations in HR pathway genes are frequently associated with ovarian, breast, and pancreatic cancer [25]. In our study, the control-based analysis revealed that germline mutations in *RAD50*, *ATM,* and *BARD1* were associated with a moderately increased risk of CRC. *RAD50*, *BARD1*, and *ATM* all function within the HR pathway, which is vital for DNA damage response and repair, thereby preserving genomic stability. Specifically, *RAD50* operates as a component of the MRE11–RAD50–NBN complex and is imperative for the repair of DNA double-strand breaks [26]. *BARD1* partners with *BRCA1* play a key role in the homologous recombination repair pathway. The *BRCA1–BARD1* tumor suppressor is an E3 ubiquitin ligase necessary for the repair of DNA double-strand breaks by HR. The *BRCA1–BARD1* complex localizes to damaged chromatin after DNA replication and catalyzes the ubiquitylation of histone *H2A* and other cellular targets [27]. *ATM*, a serine/threonine protein kinase, activates upon DNA damage, orchestrating cellular responses including DNA repair and cell cycle arrest [28]. Mutations within these genes can lead to inefficient DNA repair, causing genomic instability, which is a hallmark of cancer development. Carriers of specific gene mutations have been identified to be susceptible to other tumors, and our result implicating those gene mutations may also increase the risk of developing CRC. However, while previous studies have reported an increased risk of CRC in those *BRCA1/2* mutations carriers [23], we found no association between *BRCA1/2* mutations and CRC susceptibility in our study. The selection criteria of our study might have led to an underestimation of the potential susceptibility genes, such as *BRCA1/2*, which rank second only to the MMR genes in terms of pathogenic HR mutation prevalence. Given their high prevalence, even among healthy controls, the elevated detection of these genes in our high-risk group does not conclusively indicate an increased susceptibility to CRC.

The elevated prevalence of HR gene mutations observed in our study holds significant clinical implications. First and foremost, routine screening for HR gene mutation in CRC patients is essential to ascertain their precise prevalence and penetration in CRC. During genetic counseling, CRC patients with a family history of cancers other than CRC should be advised to undergo HR gene mutation screening. Second, HR gene mutation carriers are susceptible to other tumors such as breast cancer, pancreatic cancer, prostate cancer, etc. Thus, both CRC patients and their family member with HR gene mutations might benefit from closer surveillance of organs susceptible to these cancers. Third, investigating potential therapeutic targets remains crucial for chemoresistance of CRC [29]; the HR gene mutations as the second most prevalent ones in CRC patients, and their carrier’s demonstrated resistance to first-line chemotherapy, for which targeted therapy such as PARP inhibition might be alternative therapeutic strategy. The pathogenicity and penetration of the HR gene mutations highlighted in our study underscore the need for further clinical and foundational research. To our knowledge, the relationship between CRC's development, progression, and treatment and HR gene mutations remains largely uncharted. Further exploration through clinical trials and basic research is essential to elucidate these critical areas.

Our study highlights the unique influences of different germline mutations on both the clinical presentation and long-term prognosis of CRC patients. Broadly, patients with these germline mutations generally exhibited superior OS and PFS rates, complemented by a reduced prevalence of metastatic lymph nodes. In particular, LS patients, especially those harboring *MSH2* and *MSH6* mutation, displayed a more favorable prognosis compared to carriers of other germline mutations. This observation can likely be attributed to the inherent nature of LS-associated tumors being predominantly dMMR. Previous studies support this finding, noting that dMMR tumors typically demonstrate a superior prognosis to pMMR tumors when stages are matched [30]. Consistent with previous studies [31,32], our results demonstrated that LS patients have a significant family history of LS-associated cancer, early-onset cancer, and a propensity for multiple primary CRC. Patients with P/LP variants in HR pathway genes tended to have a higher proportion of elevated serum CEA, metastatic lymph nodes, and cancer nodules, and a lower proportion of multiple primary CRC. Compared to patients without germline mutations, HR pathway gene mutation carriers had worse PFS and OS rates, possibly due to the lack of targeted therapies for HR gene mutations in CRC [33]. FAP patients are distinguishable by their characteristic multitude of colonic adenomas [34]. Our results illustrated that FAP patients had the highest penetrance, the highest proportion of cancer nodules, *BRAF V600E* somatic mutation, and multiple primary CRC and extra-colonic cancer, as well as the worst OS and PFS.

This study has several limitations worth noting. Firstly, our detection rates for germline mutations might have been underestimated given that our PCR-based sequencing panel is not equipped to detect large rearrangements [35–37]. Secondly, the next-generation sequencing (NGS) panel had limited inherited risk-related genes, potentially missing out on detecting certain susceptibility genes. Thirdly, we focused our cancer susceptibility assessment on a select CRC population with genetic risks. As a result, CRC patients falling outside our defined 5 categories of genetic risk factors might harbor other germline mutations not addressed in our study, and we are also incapable of conducting a comparison with CRC patients without high-risk factors. Lastly, we did not examine biallelic somatic alterations, which may contribute to deviations in the molecular and clinical analysis, despite the strict filtering criteria.

In conclusion, this largest Chinese cohort study of high-risk hereditary CRC was designed as the first of its kind to cover 5 categories of genetic high-risk factors. A greatly expanded list of germline mutations were detected from the cohort, which underlie each category with distinct mutation rates and prevalence. Germline mutation screening should be performed for CRC patients with any of those genetic risk factors. CRC patients carrying different germline mutations manifested heterogeneous phenotypes in clinicopathology and long-term prognoses. In contrast to the MMR gene mutations of the LS, the study reveals for the first time at the population level that carriers of germline mutations in the HR pathway genes are significantly susceptible to CRC, implicating HR pathway gene mutations as another major contributor for increased risk of developing CRC.

## Materials and Methods

### Definitions of hereditary high-risk factors and study population

In this study, the definitions of genetic high-risk factors were defined and illustrated in Table. CRC patients with at least one of the following genetic risk factors were eligible for the study: (a) early-onset (diagnosed before age 50) CRC; (b) family history of cancer, including CRC and extra-colonic cancer including upper gastrointestinal (gastric, small bowel, and gastro-esophageal junction), gynecologic (uterine and ovarian), urogenital (bladder, renal, and prostate), breast, hepatobiliary, pancreatic, hematolymphatic, neurologic, and soft tissue cancer associated with hereditary CRC predisposition syndromes in first- and/or second-degree relatives at any age; (c) tumor IHC manifesting dMMR; (d) multiple primary CRC including synchronous and/or metachronous CRC at any age; (e) primary hereditary cancer syndrome associated extra-colonic cancer at any age. From 2015 January 1, to 2018 December 31, a total of 8,270 CRC patients received treatment at the FUSCC. A total of 2,181 CRC patients with at least one of the genetic risk factors were retrospectively enrolled in the study.

The IHC staining for MMR analysis was independently performed in the Department of Pathology at FUSCC. The majority of tumor samples were examined by IHC, although 386 tumors from patients who achieved complete response after receiving neoadjuvant therapy did not have IHC. Data including demographic information, family and medical history, pathology, and presenting symptoms were extracted from the electronic medical record. All patients were followed up as of 2021 September 30. Written informed consent was obtained from patients for genomic analysis.

### FUSCC hereditary CRC panel

We designed a multiplex polymerase chain reaction (PCR) amplification-based 38-gene FUSCC-hereditary cancer panel to detect germline mutations in eligible patients. The panel included 38 genes (*APC*, *ATM*, *ATR*, *AXIN2*, *BARD1*, *BLM*, *BMPR1A*, *BRCA1*, *BRCA2*, *BRIP1*, *CDH1*, *CDK4*, *CHEK2*, *CDKN2A*, *EPCAM*, *GALNT12*, *GREM1*, *MLH1*, *MSH2*, *MSH3*, *MSH6*, *MUTYH*, *NTHL1*, *PALB2*, *POLD1*, *POLE*, *PIK3CA*, *PMS2*, *PTEN*, *RNF43*, *RPS20*, *SMAD4*, *STK11*, *TP53*, *NBN*, *RAD50*, *RAD51C*, and *RAD51D*), 24 of which are commonly tested in multi-gene panels mentioned in NCCN guidelines (version 1. 2021) [38] and the remaining 14 genes are frequently mutated genes detected in other CRC cohorts [6,10,29,39]. The MMR genes set comprises *MLH1*, *MSH2*, *MSH6*, and *PMS2*. Meanwhile, genes associated with the HR pathway encompass *BRCA1*, *BRCA2*, *ATM*, *BARD1*, *BRIP1*, *RAD50*, *RAD51C*, *RAD51D*, *BLM*, *ATR*, *NBN*, and *PALB2*.

The gene coordinates of the coding region of each gene in hg19 are extracted from the reference genome file. The primer design uses the overlapping tile covering method by using the software “Primer 3” (version 0.4.0, https://bioinfo.ut.ee/primer3-0.4.0/) to ensure that the amplicons cover the coding region to the greatest extent. A custom library was prepared and primers were designed for all 602 coding exons (915 PCR amplicons) of these 38 genes including 180 to 280 bp of each flanking exon. Oligos were synthesized, primer droplets were prepared, and all of these droplets were pooled together to create the custom library.

Genomic DNA was purified by the use of QIAamp DNA Mini-kit (51104, QIAGEN, Germany) from white blood cells. A total of 20 to 200 ng of genomic DNA was used for PCR amplification. The primer library and a template mix that included the fragmented genomic DNA and all of the components of the PCR reaction were loaded on GeneAmp 9700 PCR (Applied Biosystems, USA) and then amplified under the following conditions: 96°C for 3 min, 17 cycles of 96°C for 30 s and 60°C for 4 min, 72°C for 4 min, and then hold at 4°C. After amplification, the amplicons from PCR droplets were purified and quality controlled using the Qubit (Thermo Fisher Scientific, USA). PCR products were subsequently used for Illumina library preparation and sequenced using an Illumina NovaSeq 5000 platform (Illumina Inc., San Diego, CA, USA).

### Germline mutation analysis

Genomic DNA was extracted from frozen peripheral lymphocytes of all enrolled patients, and the mutational spectrum was identified using the FUSCC-hereditary CRC panel. The raw data of NGS was first filtered by removing the Illumina sequencing adaptor and low-quality sequences. The remaining high-quality reads were mapped to the human reference genome (GRCh37) using the BWA aligner with the BWA-MEM algorithm and default parameters.

Germline mutations were called according to the following steps (Fig. S3). Single-nucleotide mutations were identified using the Genome Analysis ToolKit (GATK, version 4.0) [40,41] and Varscan (version 2.4.2) [42]; insertion and deletion mutations were identified based on the union results of GATK and Pindel (version 0.2.5b8) [43]. The pathogenicity of the mutations reported in Clinvar [44] with at least 2 stars was used in this study. We used the results of InterVar [45] annotation for the unreported mutations identified in this testing. We filtered the mutations using the Genome Aggregation Database (gnomAD) [46], 1000 Genomes Project [47], and the Exome Aggregation Consortium (ExAC) [48]. Only rare mutations (MAF <0.01% in 1000G 2015Aug, ExAC, or gnomAD exome database and <0.05% in the East Asian population) were selected for mutation classification. Some splicing and the stop gain mutations classified as Class 3 were upgraded to Class 4 (likely pathogenic) [49]. Only Class 4 and Class 5 (pathogenic) mutations were selected for subsequent analysis. The prevalence of P/LP variants of MMR genes and *BRCA1/2* genes in the general Chinese population was adopted from recent studies [50,51]. The prevalence of P/LP variants of other genes was re-analyzed based on the ChinaMAP reference database [52].

### Statistical analysis

Means (standard deviations) were calculated for continuous variables and percentages were calculated for categorical variables among different groups. Baseline clinical characteristics and germline mutation frequencies were compared using a 2-sided Fisher exact test. Continuous variables were compared between 2 groups by the Wilcoxon test, and the Kruskal–Wallis *H* test was used to conduct comparative statistical studies on 3 or more groups. We used logistic regression to estimate the odds ratio for PFS and OS according to different risk factors. Kaplan–Meier curves were generated, and any differences in survival were evaluated with a stratified log-rank test. Hazard ratios and confidence intervals were estimated by Cox regression analysis. All statistical analyses were performed using R (version 4.0.2), Rstudio v.1.2 software, and SPSS software (version 21.0, SPSS Inc., Chicago, USA). All statistical analyses with *P* value < 0.05 were considered statistically significant (^*^P < 0.05, ^**^P < 0.01, ^***^P < 0.001, N.S., not significant).

## Data Availability

The raw sequence data reported in this paper have been deposited in the Genome Sequence Archive in the National Genomics Data Center, China National Center for Bioinformation/Beijing Institute of Genomics, Chinese Academy of Sciences (GSA-Human: HRA004231) that are publicly accessible at https://ngdc.cncb.ac.cn/gsa-human/browse/HRA004231. Data are available from the corresponding author upon reasonable request.
