# Supplementary material for: The Largest Chinese Cohort Study Indicates Homologous Recombination Pathway Gene Mutations as Another Major Genetic Risk Factor for Colorectal Cancer with Heterogeneous Clinical Phenotypes
Source: Research (Wash D C). 2023 Oct 17;6:0249. doi: 10.34133/research.0249 (PMC10581333; doi:10.34133/research.0249)
Supplement: Supplementary 1 — Figs. S1 to S3 Tables S1 to S7 [file research.0249.f1.zip › Supplementary Tables.docx]

**Supplementary Table 1. Demographic and clinical characteristics of 2181 high-risk hereditary colorectal cancer patients.**

| Variables | Total population  (n = 2181) | dMMR  (n = 401) | Early onset  (n = 1298) | Family cancer history  (n = 803) | Extra-colonic cancer  (n = 250) | Multiple primary CRC  (n=187) |
| --- | --- | --- | --- | --- | --- | --- |
| Age (years) | 50.8 ± 12.8 (17-94) | 56.6 ± 13.9 (18-94) | 42.1 ± 6.2 (17-49) | 57 ± 12.0 (25-92) | 57.9 ± 11.9 (29-86) | 49.6 ± 12.0 (18-83) |
| < 50 | 1298 (59.5) | 134 (33.4) | 1298 (100.0) | 214 (26.7) | 75 (30.0) | 104 (55.6) |
| ≥ 50 | 883 (40.5) | 267 (66.6) | NA | 589 (73.3) | 175 (70.0) | 83 (44.4) |
| Gender |  |  |  |  |  |  |
| Male | 1215 (55.7) | 224 (55.9) | 725 (55.9) | 458 (57.0) | 98 (39.2) | 124 (66.3) |
| Female | 966 (44.3) | 177 (44.1) | 573 (44.1) | 345 (43.0) | 152 (60.8) | 63 (33.7) |
| Chronic disease |  |  |  |  |  |  |
| Presence | 272 (12.5) | 40 (10.0) | 48 (3.7) | 85 (10.6) | 27 (10.8) | 20 (10.7) |
| CEA (ng/ml) |  |  |  |  |  |  |
| < 5.2 | 1303 (59.7) | 132 (32.9) | 784 (60.4) | 351 (43.7) | 90 (36.0) | 67 (35.8) |
| CRC site |  |  |  |  |  |  |
| Right colon | 490 (22.5) | 189 (47.1) | 277 (17.5) | 171 (21.3) | 60 (24.0) | 31 (16.6) |
| Transverse colon | 144 (6.6) | 43 (10.7) | 80 (6.2) | 48 (6.0) | 22 (8.8) | 10 (5.3) |
| Left colon | 599 (27.5) | 88 (21.9) | 356 (27.4) | 237 (29.5) | 70 (28.0) | 27 (14.4) |
| Rectum | 864 (39.6) | 53 (13.2) | 586 (45.1) | 312 (38.9) | 81 (32.4) | 36 (19.3) |
| Multiple | 84 (3.8) | 28 (7.0) | 49 (3.8) | 35 (4.4) | 17 (6.8) | 83 (44.4) |
| Pathological type |  |  |  |  |  |  |
| Adenocarcinoma | 1850 (84.8) | 305 (76.1) | 1108 (85.4) | 695 (86.6) | 217 (86.8) | 154 (82.4) |
| Mucinous | 254 (11.6) | 84 (20.9) | 131 (10.1) | 88 (11.0) | 27 (10.8) | 24 (12.8) |
| Signer ring cell carcinoma | 77 (3.6) | 12 (3.0) | 59 (4.5) | 20 (2.5) | 6 (2.4) | 9 (4.8) |
| Differentiation grade |  |  |  |  |  |  |
| Well | 112 (5.1) | 11 (2.7) | 69 (5.3) | 42 (5.2) | 21 (8.4) | 10 (5.3) |
| Moderately | 1394 (63.9) | 209 (52.1) | 824 (63.5) | 554 (69.0) | 163 (65.2) | 118 (63.1) |
| Poorly | 675 (31.0) | 181 (45.1) | 405 (31.2) | 207 (25.8) | 66 (26.4) | 59 (31.6) |
| Cancerous node |  |  |  |  |  |  |
| Occurrence | 322 (14.8) | 26 (6.5) | 210 (16.2) | 122 (15.2) | 34 (13.6) | 17 (9.1) |
| Vascular invasion |  |  |  |  |  |  |
| Occurrence | 597 (27.4) | 88 (21.9) | 393 (30.3) | 194 (24.2) | 62 (24.8) | 44 (23.5) |
| Perineural invasion |  |  |  |  |  |  |
| Occurrence | 617 (28.3) | 80 (20.0) | 406 (31.3) | 206 (25.7) | 64 (25.6) | 42 (22.5) |
| TNM stage |  |  |  |  |  |  |
| 0 | 57 (2.6) | 2 (0.5) | 37 (2.9) | 21 (2.6) | 13 (5.2) | 6 (3.2) |
| I | 409 (18.8) | 69 (17.2) | 231 (17.8) | 169 (21.0) | 56 (22.4) | 57 (30.5) |
| II | 711 (32.6) | 220 (54.9) | 362 (27.9) | 266 (33.1) | 79 (31.6) | 54 (28.9) |
| III | 770 (35.3) | 95 (23.7) | 491 (37.8) | 274 (34.1) | 86 (34.4) | 52 (27.8) |
| IV | 234 (10.7) | 15 (3.7) | 177 (13.6) | 73 (9.1) | 16 (6.4) | 18 (9.6) |
| IHC MMR |  |  |  |  |  |  |
| Deficiency | 401 (18.4) | 401 (100.0) | 134 (10.3) | 93 (11.6) | 45 (18.0) | 62 (33.2) |
| Proficiency | 1394 (63.9) | NA | 889 (68.5) | 569 (70.9) | 169 (67.6) | 100 (53.5) |
| Not applicable | 386 (17.7) | NA | 275 (21.2) | 141 (17.6) | 36 (14.4) | 25 (13.4) |
| *RAS* genotype |  |  |  |  |  |  |
| Wild type | 478 (21.9) | 149 (37.2) | 301 (23.2) | 132 (16.4) | 54 (21.6) | 49 (26.2) |
| Mutant type | 402 (18.4) | 93 (23.2) | 242 (18.6) | 125 (15.6) | 45 (18.0) | 39 (20.9) |
| Not applicable | 1301 (59.7) | 159 (39.7) | 755 (58.2) | 546 (68.0) | 151 (60.4) | 99 (52.9) |
| *BRAF* genotype |  |  |  |  |  |  |
| Wild type | 817 (37.5) | 211 (52.6) | 514 (39.6) | 244 (30.4) | 91 (36.4) | 84 (44.9) |
| Mutant type | 63(2.9) | 31 (7.7) | 29 (2.2) | 13 (1.6) | 8 (3.2) | 4 (2.1) |
| Not applicable | 1301 (59.6) | 159 (39.7) | 755 (58.2) | 546 (68.0) | 151 (60.4) | 99 (52.9) |
| Microsatellite |  |  |  |  |  |  |
| Stable | 77 (3.5) | 6 (1.5) | 52 (4.0) | 30 (3.7) | 9 (3.6) | 6 (3.2) |
| Instability-low | 4 (0.2) | 2 (0.5) | 3 (0.2) | 1 (0.1) | 1 (0.4) | NA |
| Instability-high | 82 (3.8) | 65 (16.2) | 31 (2.4) | 20 (2.5) | 14 (5.6) | 19 (10.2) |
| Not applicable | 2018 (92.5) | 328 (81.8) | 1212 (93.4) | 752 (93.6) | 226 (90.4) | 162 (86.6) |
| Multiple primary CRC |  |  |  |  |  |  |
| Occurrence | 187 (8.6) | 62 (15.5) | 104 (8.0) | 80 (10.0) | 45 (18.0) | 187 (100.0) |
| Extra-colonic cancer |  |  |  |  |  |  |
| Occurrence | 250 (11.5) | 45 (11.2) | 75 (5.8) | 103 (12.8) | 250 (100.0) | 45 (24.1) |

**Supplementary Table 2**. Pathogenic and likely pathogenic mutations list.

| Patient ID | Genes | Transcript | Nuclotide Change | Amino Acid Change | Function |
| --- | --- | --- | --- | --- | --- |
| CB140411 | MSH2 | NM_000251 | c.T518C | p.L173P | Likely pathogenic |
| CB150210 | MSH2 | NM_000251 | c.G1090T | p.E364X | Pathogenic |
| CB150323 | MSH2 | NM_000251 | c.G894T | p.Q298H | Likely pathogenic |
| CB150405 | MSH2 | NM_000251 | c.A1121G | p.Q374R | Likely pathogenic |
| CB150569 | MSH2 | NM_000251 | c.1760_1805AACACATCATTGAGTGTCTGCATTGGTTCTACATAGCCTGTATAAA | Splice_Site | Likely pathogenic |
| CB150752 | MSH2 | NM_000251 | c.C2038T | p.R680X | Pathogenic |
| CB150914 | MSH2 | NM_000251 | c.C1528T | p.Q510X | Pathogenic |
| CB151252 | MSH2 | NM_000251 | c.1510+1G>A | Splice_Site | Likely pathogenic |
| CB151265 | MSH2 | NM_000251 | c.C1165T | p.R389X | Pathogenic |
| CB151667 | MSH2 | NM_000251 | c.T929C | p.L310P | Pathogenic |
| CB151697 | MSH2 | NM_000251 | c.T929C | p.L310P | Pathogenic |
| CB151772 | MSH2 | NM_000251 | c.T929C | p.L310P | Pathogenic |
| CB160276 | MSH2 | NM_000251 | c.C1009T | p.Q337X | Pathogenic |
| CB160341 | MSH2 | NM_000251 | c.G1738T | p.E580X | Pathogenic |
| CB160679 | MSH2 | NM_000251 | c.T518C | p.L173P | Likely pathogenic |
| CB160734 | MSH2 | NM_000251 | c.547delC | p.Q183fs | Likely pathogenic |
| CB160992 | MSH2 | NM_000251 | c.1786_1788del | p.596_596del | Pathogenic |
| CB161034 | MSH2 | NM_000251 | c.T929C | p.L310P | Pathogenic |
| CB161188 | MSH2 | NM_000251 | c.A557G | p.N186S | Likely pathogenic |
| CB161193 | MSH2 | NM_000251 | c.T929C | p.L310P | Pathogenic |
| CB161384 | MSH2 | NM_000251 | c.C1183T | p.Q395X | Pathogenic |
| CB161437 | MSH2 | NM_000251 | c.A1077T | p.R359S | Pathogenic |
| CB161522 | MSH2 | NM_000251 | c.1511-2A>G | Splice_Site | Pathogenic |
| CB161572 | MSH2 | NM_000251 | c.1386+1G>T | Splice_Site | Likely pathogenic |
| CB161686 | MSH2 | NM_000251 | c.A1121G | p.Q374R | Likely pathogenic |
| CB161723 | MSH2 | NM_000251 | c.T599A | p.V200D | Pathogenic |
| CB161917 | MSH2 | NM_000251 | c.G859T | p.G287X | Pathogenic |
| CB170185 | MSH2 | NM_000251 | c.C2131T | p.R711X | Pathogenic |
| CB170299 | MSH2 | NM_000251 | c.350dupG | p.W117fs | Likely pathogenic |
| CB170345 | MSH2 | NM_000251 | c.T1373G | p.L458X | Pathogenic |
| CB170689 | MSH2 | NM_000251 | c.C1009T | p.Q337X | Pathogenic |
| CB171054 | MSH2 | NM_000251 | c.183dupG | p.Q61fs | Pathogenic |
| CB171314 | MSH2 | NM_000251 | c.G2039C | p.R680P | Likely pathogenic |
| CB171888 | MSH2 | NM_000251 | c.350dupG | p.W117fs | Likely pathogenic |
| CB172162 | MSH2 | NM_000251 | c.2006-1G>A | Splice_Site | Likely pathogenic |
| CB180006 | MSH2 | NM_000251 | c.164delG | p.R55fs | Pathogenic |
| CB180202 | MSH2 | NM_000251 | c.C1042T | p.Q348X | Pathogenic |
| CB180253 | MSH2 | NM_000251 | c.T518C | p.L173P | Likely pathogenic |
| CB180478 | MSH2 | NM_000251 | c.1786_1788del | p.596_596del | Pathogenic |
| CB180755 | MSH2 | NM_000251 | c.1567delT | p.F523fs | Likely pathogenic |
| CB180823 | MSH2 | NM_000251 | c.943-1G>C | Splice_Site | Likely pathogenic |
| CB181136 | MSH2 | NM_000251 | c.C547T | p.Q183X | Pathogenic |
| CB181171 | MSH2 | NM_000251 | c.T929A | p.L310H | Likely pathogenic |
| CB181407 | MSH2 | NM_000251 | c.C1165T | p.R389X | Pathogenic |
| CB181613 | MSH2 | NM_000251 | c.T518C | p.L173P | Likely pathogenic |
| CB181811 | MSH2 | NM_000251 | c.C2027A | p.S676X | Pathogenic |
| CB182655 | MSH2 | NM_000251 | c.C1216T | p.R406X | Pathogenic |
| CB190687 | MSH2 | NM_000251 | c.G1012C | p.G338R | Likely pathogenic |
| CB190729 | MSH2 | NM_000251 | c.A244T | p.K82X | Pathogenic |
| CB190748 | MSH2 | NM_000251 | c.366+1G>T | Splice_Site | Likely pathogenic |
| CB190932 | MSH2 | NM_000251 | c.1115_1116del | p.L372fs | Likely pathogenic |
| CB191112 | MSH2 | NM_000251 | c.G859T | p.G287X | Pathogenic |
| CB191116 | MSH2 | NM_000251 | c.C1165T | p.R389X | Pathogenic |
| CB191374 | MSH2 | NM_000251 | c.182delA | p.Q61fs | Pathogenic |
| CB191437 | MSH2 | NM_000251 | c.942+2T>A | Splice_Site | Likely pathogenic |
| CB191575 | MSH2 | NM_000251 | c.C2038T | p.R680X | Pathogenic |
| CB191794 | MSH2 | NM_000251 | c.976_979del | p.L326fs | Likely pathogenic |
| CB192236 | MSH2 | NM_000251 | c.C1861T | p.R621X | Pathogenic |
| CB192369 | MSH2 | NM_000251 | c.942+1->T | Splice_Site | Likely pathogenic |
| CB192541 | MSH2 | NM_000251 | c.1990dupT | p.M663fs | Likely pathogenic |
| CB192567 | MSH2 | NM_000251 | c.G942T | p.Q314H | Likely pathogenic |
| CB192707 | MSH2 | NM_000251 | c.C652T | p.Q218X | Pathogenic |
| CB200257 | MSH2 | NM_000251 | c.T518C | p.L173P | Likely pathogenic |
| CB150233 | MSH3 | NM_002439 | c.3001_3053GGTGAAATCCTTAACCCAGTTTGTCACCCATTATCCGCCAGTTTGTGAACTAG | Splice_Site | Likely pathogenic |
| CB150272 | MSH3 | NM_002439 | c.3060delT | p.N1020fs | Likely pathogenic |
| CB150410 | MSH3 | NM_002439 | c.3053dupA | p.E1018fs | Likely pathogenic |
| CB150460 | MSH3 | NM_002439 | c.3001_3014GTGAAATCCTAAAA | Splice_Site | Likely pathogenic |
| CB151142 | MSH3 | NM_002439 | c.3053delA | p.E1018fs | Likely pathogenic |
| CB151356 | MSH3 | NM_002439 | c.3053delA | p.E1018fs | Likely pathogenic |
| CB160418 | MSH3 | NM_002439 | c.3084delC | p.Y1028fs | Likely pathogenic |
| CB161480 | MSH3 | NM_002439 | c.3069delC | p.H1023fs | Likely pathogenic |
| CB161870 | MSH3 | NM_002439 | c.3053delA | p.E1018fs | Likely pathogenic |
| CB171005 | MSH3 | NM_002439 | c.3053delA | p.E1018fs | Likely pathogenic |
| CB181278 | MSH3 | NM_002439 | c.1557dupC | p.L519fs | Likely pathogenic |
| CB181867 | MSH3 | NM_002439 | c.1764-2A>G | Splice_Site | Likely pathogenic |
| CB182072 | MSH3 | NM_002439 | c.3235dupC | p.V1078fs | Likely pathogenic |
| CB191062 | MSH3 | NM_002439 | c.C3171G | p.Y1057X | Pathogenic |
| CB150502 | MUTYH | NM_001128425 | c.C55T | p.R19X | Pathogenic |
| CB150981 | MUTYH | NM_001128425 | c.C55T | p.R19X | Pathogenic |
| CB161248 | MUTYH | NM_001128425 | c.C55T | p.R19X | Pathogenic |
| CB161481 | MUTYH | NM_001128425 | c.G467A | p.W156X | Pathogenic |
| CB170394 | MUTYH | NM_001128425 | c.G467A | p.W156X | Pathogenic |
| CB170524 | MUTYH | NM_001128425 | c.C55T | p.R19X | Pathogenic |
| CB170651 | MUTYH | NM_001128425 | c.C799T | p.Q267X | Pathogenic |
| CB170686 | MUTYH | NM_001128425 | c.G467A | p.W156X | Pathogenic |
| CB170784 | MUTYH | NM_001128425 | c.C799T | p.Q267X | Pathogenic |
| CB171003 | MUTYH | NM_001128425 | c.C55T | p.R19X | Pathogenic |
| CB171334 | MUTYH | NM_001128425 | c.C721T | p.R241W | Likely pathogenic |
| CB172138 | MUTYH | NM_001128425 | c.G467A | p.W156X | Pathogenic |
| CB181094 | MUTYH | NM_001128425 | c.C55T | p.R19X | Pathogenic |
| CB182520 | MUTYH | NM_001128425 | c.C1301G | p.T434R | Likely pathogenic |
| CB191308 | MUTYH | NM_001128425 | c.C671T | p.A224V | Likely pathogenic |
| CB150776 | NBN | NM_002485 | c.2185-2A>C | Splice_Site | Likely pathogenic |
| CB160583 | NTHL1 | NM_002528 | c.450_457del | p.G150fs | Likely pathogenic |
| CB182238 | NTHL1 | NM_002528 | c.353dupA | p.K118fs | Likely pathogenic |
| CB170048 | PALB2 | NM_024675 | c.3114-1G>A | Splice_Site | Likely pathogenic |
| CB181366 | PALB2 | NM_024675 | c.2926_2928del | p.976_976del | Pathogenic |
| CB171845 | PIK3CA | NM_006218 | c.2663dupA | p.E888fs | Likely pathogenic |
| CB181832 | PIK3CA | NM_006218 | c.G2309A | p.R770Q | Likely pathogenic |
| CB190675 | POLD1 | NM_002691 | c.C2467A | p.R823S | Likely pathogenic |
| CB151635 | RAD50 | NM_005732 | c.2157dupA | p.L719fs | Pathogenic |
| CB161098 | RAD50 | NM_005732 | c.C778T | p.H260Y | Likely pathogenic |
| CB161119 | RAD50 | NM_005732 | c.C2141A | p.S714X | Pathogenic |
| CB161147 | RAD50 | NM_005732 | c.C778T | p.H260Y | Likely pathogenic |
| CB161552 | RAD50 | NM_005732 | c.C2141A | p.S714X | Pathogenic |
| CB170048 | RAD50 | NM_005732 | c.C2141A | p.S714X | Pathogenic |
| CB170057 | RAD50 | NM_005732 | c.C2141A | p.S714X | Pathogenic |
| CB170588 | RAD50 | NM_005732 | c.C2141A | p.S714X | Pathogenic |
| CB170975 | RAD50 | NM_005732 | c.C2141A | p.S714X | Pathogenic |
| CB170980 | RAD50 | NM_005732 | c.C2141A | p.S714X | Pathogenic |
| CB171205 | RAD50 | NM_005732 | c.C2141A | p.S714X | Pathogenic |
| CB171264 | RAD50 | NM_005732 | c.2157dupA | p.L719fs | Pathogenic |
| CB180161 | RAD50 | NM_005732 | c.C412T | p.R138X | Pathogenic |
| CB180374 | RAD50 | NM_005732 | c.2794delA | p.K932fs | Pathogenic |
| CB181235 | RAD50 | NM_005732 | c.2165_2166insT | p.K722fs | Pathogenic |
| CB182117 | RAD50 | NM_005732 | c.2846_2848del | p.949_950del | Pathogenic |
| CB182417 | RAD50 | NM_005732 | c.1200_1203del | p.V400fs | Pathogenic |
| CB150220 | RAD51C | NM_058216 | c.A758T | p.D253V | Likely pathogenic |
| CB160693 | RAD51C | NM_058216 | c.390dupA | p.G130fs | Pathogenic |
| CB171972 | RAD51C | NM_058216 | c.905-2A>C | Splice_Site | Likely pathogenic |
| CB180465 | RAD51C | NM_058216 | c.A476T | p.D159V | Likely pathogenic |
| CB190073 | RNF43 | NM_017763 | c.C988T | p.R330X | Likely pathogenic |
| CB161127 | SMAD4 | NM_005359 | c.G265T | p.G89X | Pathogenic |
| CB182463 | STK11 | NM_000455 | c.G652C | p.A218P | Likely pathogenic |
| CB150053 | TP53 | NM_000546 | c.G31C | p.E11Q | Likely pathogenic |
| CB150522 | TP53 | NM_000546 | c.93_96CTGG | Splice_Site | Likely pathogenic |
| CB160731 | TP53 | NM_000546 | c.C604T | p.R202C | Likely pathogenic |
| CB160755 | TP53 | NM_000546 | c.C541T | p.R181C | Likely pathogenic |
| CB161064 | TP53 | NM_000546 | c.G814T | p.V272L | Likely pathogenic |
| CB161423 | TP53 | NM_000546 | c.G818T | p.R273L | Pathogenic |
| CB161497 | TP53 | NM_000546 | c.G1015A | p.E339K | Likely pathogenic |
| CB161620 | TP53 | NM_000546 | c.G733A | p.G245S | Pathogenic |
| CB182579 | TP53 | NM_000546 | c.375+1G>C | Splice_Site | Likely pathogenic |
| CB150287 | RAD51D | NM_001142571 | c.331_332insTA | p.K111fs | Likely pathogenic |
| CB150906 | RAD51D | NM_001142571 | c.331_332insTA | p.K111fs | Likely pathogenic |
| CB151162 | RAD51D | NM_001142571 | c.331_332insTA | p.K111fs | Likely pathogenic |
| CB161022 | RAD51D | NM_001142571 | c.331_332insTA | p.K111fs | Likely pathogenic |
| CB161191 | RAD51D | NM_001142571 | c.331_332insTA | p.K111fs | Likely pathogenic |
| CB161424 | RAD51D | NM_001142571 | c.331_332insTA | p.K111fs | Likely pathogenic |
| CB182102 | RAD51D | NM_001142571 | c.331_332insTA | p.K111fs | Likely pathogenic |
| CB150212 | POLE | NM_006231 | c.6695delT | p.M2232fs | Likely pathogenic |
| CB150218 | POLE | NM_006231 | c.6702_6703del | p.V2234fs | Likely pathogenic |
| CB150220 | POLE | NM_006231 | c.G2881T | p.E961X | Pathogenic |
| CB150366 | POLE | NM_006231 | c.G2881T | p.E961X | Pathogenic |
| CB150970 | POLE | NM_006231 | c.6715_6747AGGAGGCCAGGCTGAGCCGAGGCAGATGAGGGA | Splice_Site | Likely pathogenic |
| CB151035 | POLE | NM_006231 | c.6696delG | p.M2232fs | Likely pathogenic |
| CB160336 | POLE | NM_006231 | c.6747+1G>- | Splice_Site | Likely pathogenic |
| CB160818 | POLE | NM_006231 | c.3796-2A>C | Splice_Site | Likely pathogenic |
| CB161054 | POLE | NM_006231 | c.6745delC | p.Q2249fs | Likely pathogenic |
| CB161191 | POLE | NM_006231 | c.G1957A | p.A653T | Likely pathogenic |
| CB161802 | POLE | NM_006231 | c.6123_6136del | p.V2041fs | Likely pathogenic |
| CB161932 | POLE | NM_006231 | c.6695delT | p.M2232fs | Likely pathogenic |
| CB170784 | POLE | NM_006231 | c.G2308A | p.G770R | Likely pathogenic |
| CB171594 | POLE | NM_006231 | c.G2881T | p.E961X | Pathogenic |
| CB180305 | POLE | NM_006231 | c.6698delC | p.P2233fs | Likely pathogenic |
| CB181474 | POLE | NM_006231 | c.C5229G | p.H1743Q | Likely pathogenic |
| CB182306 | POLE | NM_006231 | c.6698delC | p.P2233fs | Likely pathogenic |
| CB151868 | PTEN | NM_001304717 | c.G1438A | p.E480K | Likely pathogenic |
| CB150398 | PTEN | NM_001304717 | c.-366T>C | Splice_Site | Likely pathogenic |
| CB150441 | PTEN | NM_001304717 | c.-366T>C | Splice_Site | Likely pathogenic |
| CB151697 | PTEN | NM_001304717 | c.-366T>C | Splice_Site | Likely pathogenic |
| CB140591 | PMS2 | NM_000535 | c.164-1G>A | Splice_Site | Likely pathogenic |
| CB151366 | PMS2 | NM_000535 | c.A46T | p.K16X | Pathogenic |
| CB160655 | PMS2 | NM_000535 | c.C595T | p.R199C | Likely pathogenic |
| CB161273 | PMS2 | NM_000535 | c.A1G | p.M1V | Likely pathogenic |
| CB161562 | PMS2 | NM_000535 | c.1053delG | p.L351fs | Pathogenic |
| CB161768 | PMS2 | NM_000535 | c.1579_1580del | p.R527fs | Pathogenic |
| CB161990 | PMS2 | NM_000535 | c.C943T | p.R315X | Pathogenic |
| CB171797 | PMS2 | NM_000535 | c.538-2A>G | Splice_Site | Likely pathogenic |
| CB180121 | PMS2 | NM_000535 | c.A1738T | p.K580X | Pathogenic |
| CB180545 | PMS2 | NM_000535 | c.164-1G>A | Splice_Site | Likely pathogenic |
| CB180680 | PMS2 | NM_000535 | c.1119_1122del | p.S373fs | Pathogenic |
| CB180831 | PMS2 | NM_000535 | c.1997_1998del | p.K666fs | Likely pathogenic |
| CB182842 | PMS2 | NM_000535 | c.861_864del | p.R287fs | Pathogenic |
| CB190724 | PMS2 | NM_000535 | c.C1882T | p.R628X | Pathogenic |
| CB190944 | PMS2 | NM_000535 | c.C2444T | p.S815L | Likely pathogenic |
| CB191958 | PMS2 | NM_000535 | c.1053delG | p.L351fs | Pathogenic |
| CB200321 | PMS2 | NM_000535 | c.A1G | p.M1V | Likely pathogenic |
| CB200586 | PMS2 | NM_000535 | c.A1004G | p.N335S | Likely pathogenic |
| CB150178 | MSH6 | NM_000179 | c.1561_1562del | p.T521fs | Likely pathogenic |
| CB150366 | MSH6 | NM_000179 | c.3254delC | p.T1085fs | Pathogenic |
| CB150421 | MSH6 | NM_000179 | c.3065delA | p.E1022fs | Likely pathogenic |
| CB150982 | MSH6 | NM_000179 | c.G3610C | p.A1204P | Likely pathogenic |
| CB151264 | MSH6 | NM_000179 | c.C3260G | p.P1087R | Likely pathogenic |
| CB151288 | MSH6 | NM_000179 | c.G2562T | p.K854N | Likely pathogenic |
| CB151491 | MSH6 | NM_000179 | c.3306dupT | p.T1102fs | Pathogenic |
| CB151758 | MSH6 | NM_000179 | c.627+2T>A | Splice_Site | Likely pathogenic |
| CB151944 | MSH6 | NM_000179 | c.4001+2_4001+26del | Splice_Site | Likely pathogenic |
| CB160442 | MSH6 | NM_000179 | c.C642G | p.Y214X | Pathogenic |
| CB161229 | MSH6 | NM_000179 | c.G3725A | p.R1242H | Likely pathogenic |
| CB161800 | MSH6 | NM_000179 | c.3992_4001del | p.R1331fs | Likely pathogenic |
| CB161813 | MSH6 | NM_000179 | c.C3226T | p.R1076C | Likely pathogenic |
| CB170020 | MSH6 | NM_000179 | c.C3226G | p.R1076G | Likely pathogenic |
| CB170233 | MSH6 | NM_000179 | c.3254delC | p.T1085fs | Pathogenic |
| CB170251 | MSH6 | NM_000179 | c.742delC | p.R248fs | Pathogenic |
| CB170263 | MSH6 | NM_000179 | c.C3202T | p.R1068X | Pathogenic |
| CB170621 | MSH6 | NM_000179 | c.2106delA | p.S702fs | Likely pathogenic |
| CB170990 | MSH6 | NM_000179 | c.A3944C | p.K1315T | Likely pathogenic |
| CB171348 | MSH6 | NM_000179 | c.C3202T | p.R1068X | Pathogenic |
| CB171687 | MSH6 | NM_000179 | c.1571dupA | p.Y524_S525delinsX | Pathogenic |
| CB171739 | MSH6 | NM_000179 | c.C3226T | p.R1076C | Likely pathogenic |
| CB171739 | MSH6 | NM_000179 | c.3131dupA | p.Y1044_K1045delinsX | Likely pathogenic |
| CB171784 | MSH6 | NM_000179 | c.2292delT | p.T764fs | Likely pathogenic |
| CB171816 | MSH6 | NM_000179 | c.3445_3447del | p.1149_1149del | Pathogenic |
| CB172210 | MSH6 | NM_000179 | c.C3851T | p.T1284M | Likely pathogenic |
| CB180013 | MSH6 | NM_000179 | c.3831dupC | p.D1277fs | Likely pathogenic |
| CB180160 | MSH6 | NM_000179 | c.G2419C | p.E807Q | Likely pathogenic |
| CB180201 | MSH6 | NM_000179 | c.3254delC | p.T1085fs | Pathogenic |
| CB180206 | MSH6 | NM_000179 | c.T3664A | p.F1222I | Likely pathogenic |
| CB180327 | MSH6 | NM_000179 | c.C3132G | p.Y1044X | Pathogenic |
| CB180572 | MSH6 | NM_000179 | c.3254dupC | p.T1085fs | Pathogenic |
| CB181697 | MSH6 | NM_000179 | c.651dupT | p.D217fs | Pathogenic |
| CB181735 | MSH6 | NM_000179 | c.C3103T | p.R1035X | Pathogenic |
| CB182312 | MSH6 | NM_000179 | c.G3788A | p.R1263H | Likely pathogenic |
| CB190418 | MSH6 | NM_000179 | c.G2372C | p.R791P | Likely pathogenic |
| CB190692 | MSH6 | NM_000179 | c.G616T | p.E206X | Pathogenic |
| CB190724 | MSH6 | NM_000179 | c.2106delA | p.S702fs | Likely pathogenic |
| CB190750 | MSH6 | NM_000179 | c.T1767A | p.Y589X | Pathogenic |
| CB190951 | MSH6 | NM_000179 | c.1631dupA | p.E544fs | Likely pathogenic |
| CB190978 | MSH6 | NM_000179 | c.1339dupC | p.E446fs | Likely pathogenic |
| CB190994 | MSH6 | NM_000179 | c.3959_3960insAAGA | p.A1320fs | Pathogenic |
| CB191123 | MSH6 | NM_000179 | c.3677_3678insAATAGCAAATGCAGTTGTTAA | p.A1226delinsAIANAVVK | Pathogenic |
| CB191226 | MSH6 | NM_000179 | c.1381delT | p.F461fs | Likely pathogenic |
| CB191649 | MSH6 | NM_000179 | c.C3986A | p.S1329X | Pathogenic |
| CB191709 | MSH6 | NM_000179 | c.C2194T | p.R732X | Pathogenic |
| CB191807 | MSH6 | NM_000179 | c.753dupA | p.I251fs | Likely pathogenic |
| CB192414 | MSH6 | NM_000179 | c.3445_3447del | p.1149_1149del | Pathogenic |
| CB200586 | MSH6 | NM_000179 | c.3677_3678insAATAGCAAATGCAGTTGTTAA | p.A1226delinsAIANAVVK | Pathogenic |
| CB160766 | RAD51D | NM_001142571 | c.C298T | p.R100X | Likely pathogenic |
| CB161280 | RAD51D | NM_001142571 | c.C184T | p.Q62X | Likely pathogenic |
| CB150398 | APC | NM_000038 | c.T875A | p.L292X | Pathogenic |
| CB160565 | APC | NM_000038 | c.C8161G | p.R2721G | Likely pathogenic |
| CB170531 | APC | NM_000038 | c.T3542G | p.L1181X | Likely pathogenic |
| CB171708 | APC | NM_000038 | c.C6970T | p.P2324 | Likely pathogenic |
| CB172087 | APC | NM_001127511 | c.G67C | p.G23R | Likely pathogenic |
| CB180122 | APC | NM_000038 | c.C694T | p.R232X | Pathogenic |
| CB180354 | APC | NM_000038 | c.G2033A | p.S678N | Likely pathogenic |
| CB180711 | APC | NM_000038 | c.1548+1G>A | Splice_Site | Pathogenic |
| CB181196 | APC | NM_000038 | c.A1870G | p.S624G |  |
| CB182112 | APC | NM_000038 | c.C6970T | p.P2324S |  |
| CB150489 | APC | NM_000038 | c.1651_1655del | p.L551fs |  |
| CB150877 | APC | NM_000038 | c.1651_1655del | p.L551fs |  |
| CB151443 | APC | NM_000038 | c.531+1_531+4del | Splice_Site |  |
| CB160089 | APC | NM_000038 | c.3921_3925del | p.I1307fs |  |
| CB160262 | APC | NM_000038 | c.1865_1873del | p.622_625del |  |
| CB170306 | APC | NM_000038 | c.2483delC | p.T828fs |  |
| CB170335 | APC | NM_000038 | c.3180_3184del | p.I1060fs |  |
| CB151297 | ATM | NM_000051 | c.G6368A | p.S2123N | Likely pathogenic |
| CB170578 | ATM | NM_000051 | c.C5697A | p.C1899X | Pathogenic |
| CB172221 | ATM | NM_000051 | c.C2486T | p.P829L | Likely pathogenic |
| CB182221 | ATM | NM_000051 | c.C67T | p.R23X | Pathogenic |
| CB200019 | ATM | NM_000051 | c.C9139T | p.R3047X | Pathogenic |
| CB150026 | ATM | NM_000051 | c.85delA | p.K29fs | Likely pathogenic |
| CB150212 | ATM | NM_000051 | c.609_610del | p.D203fs | Likely pathogenic |
| CB150233 | ATM | NM_000051 | c.3482delT | p.V1161fs | Pathogenic |
| CB150249 | ATM | NM_000051 | c.85delA | p.K29fs | Likely pathogenic |
| CB150255 | ATM | NM_000051 | c.88dupT | p.K29fs | Likely pathogenic |
| CB150310 | ATM | NM_000051 | c.8030delA | p.Y2677fs | Likely pathogenic |
| CB150456 | ATM | NM_000051 | c.89_91del | p.30_31del | Pathogenic |
| CB151020 | ATM | NM_000051 | c.85delA | p.K29fs | Likely pathogenic |
| CB151067 | ATM | NM_000051 | c.3436_3437insAATTTCTTTTTAAGTCCCATAGTGCTGAGAACCCTGAAACTTTGGATGAAATTTATAATTGATTGTTAAACATTTACATTTTACATTACATTTTTTTTA | p.E1146delinsEFLFKSHSAENPETLDEIYNX | Likely pathogenic |
| CB151087 | ATM | NM_000051 | c.3506dupA | p.E1169fs | Pathogenic |
| CB151399 | ATM | NM_000051 | c.2282_2283del | p.T761fs | Pathogenic |
| CB160418 | ATM | NM_000051 | c.85delA | p.K29fs | Likely pathogenic |
| CB161027 | ATM | NM_000051 | c.6310_6311insGGAG | p.W2104fs | Likely pathogenic |
| CB161144 | ATM | NM_000051 | c.3458delT | p.V1153fs | Likely pathogenic |
| CB161235 | ATM | NM_000051 | c.497_535AATTGTTCTCTGTGTACTTCAGGCTCAATATGAAAACTA | Splice_Site | Likely pathogenic |
| CB161701 | ATM | NM_000051 | c.3609delT | p.Y1203X | Pathogenic |
| CB170104 | ATM | NM_000051 | c.85delA | p.K29fs | Likely pathogenic |
| CB170316 | ATM | NM_000051 | c.3436_3437insTTTTTTTTTTAATTTCTTTTTAAGTCCCATAGTGCTGAGAACCCTGAAACTTTGGATGAAATTTATAATTGATTGTTAAACATTTACATTTTACATTACATT | p.E1146delinsVFFLISFX | Likely pathogenic |
| CB180305 | ATM | NM_000051 | c.612delA | p.G204fs | Likely pathogenic |
| CB180516 | ATM | NM_000051 | c.3403_3467TCCCATAGTGCTGAGAACCCTGAAACTTTGGATGAAATTTATAATAGAAAATATGTTTTAATTAA | Splice_Site | Likely pathogenic |
| CB180746 | ATM | NM_000051 | c.85delA | p.K29fs | Likely pathogenic |
| CB180778 | ATM | NM_000051 | c.3404delC | p.S1135fs | Likely pathogenic |
| CB181028 | ATM | NM_000051 | c.3436_3437insAATTTCTTTTTAAGTCCCATAGTGCTGAGAACCCTGAAACTTTGGATGAAATTTATAATTGATTGTTAAACATTTACATTTTACATTACATTTTTTTTTA | p.E1146_I1147delinsEFLFKSHSAENPETLDEIYNX | Likely pathogenic |
| CB181271 | ATM | NM_000051 | c.3436_3437insAATTTCTTTTTAAGTCCCATAGTGCTGAGAACCCTGAAACTTTGGATGAAATTTATAATTGATTGTTAAACATTTACATTTTACATTACATTTTTTTTTA | p.E1146_I1147delinsEFLFKSHSAENPETLDEIYNX | Likely pathogenic |
| CB161235 | ATR | NM_001184 | c.C115T | p.Q39X | Pathogenic |
| CB170332 | ATR | NM_001184 | c.G1953A | p.W651X | Pathogenic |
| CB151373 | ATR | NM_001184 | c.1149delT | p.L383fs | Likely pathogenic |
| CB160015 | ATR | NM_001184 | c.4246dupT | p.S1416fs | Likely pathogenic |
| CB162162 | ATR | NM_001184 | c.1139dupT | p.L380fs | Likely pathogenic |
| CB171232 | ATR | NM_001184 | c.1139dupT | p.L380fs | Likely pathogenic |
| CB182620 | ATR | NM_001184 | c.1139dupT | p.L380fs | Likely pathogenic |
| CB160329 | AXIN2 | NM_004655 | c.C1658A | p.S553X | Pathogenic |
| CB181061 | AXIN2 | NM_004655 | c.1060-1G>T | Splice_Site | Likely pathogenic |
| CB180374 | AXIN2 | NM_004655 | c.1442_1491del | p.L481fs | Likely pathogenic |
| CB180741 | AXIN2 | NM_004655 | c.1416_1421del | p.472_474del | Pathogenic |
| CB140933 | BARD1 | NM_000465 | c.C242A | p.T81N | Likely pathogenic |
| CB141189 | BARD1 | NM_000465 | c.C242A | p.T81N | Likely pathogenic |
| CB150366 | BARD1 | NM_000465 | c.C242A | p.T81N | Likely pathogenic |
| CB160325 | BARD1 | NM_000465 | c.C242A | p.T81N | Likely pathogenic |
| CB160559 | BARD1 | NM_000465 | c.T254A | p.V85E | Likely pathogenic |
| CB140314 | BARD1 | NM_000465 | c.1051_1092CGGTGCCCTCAGAAAATATA | Splice_Site | Likely pathogenic |
| CB150409 | BARD1 | NM_000465 | c.1051_1092CGGTGCCCTCAGAAAATATA | Splice_Site | Likely pathogenic |
| CB150463 | BARD1 | NM_000465 | c.1067_1092TATA | Splice_Site | Likely pathogenic |
| CB150505 | BARD1 | NM_000465 | c.1067dupA | p.N356fs | Likely pathogenic |
| CB151545 | BARD1 | NM_000465 | c.1006delT | p.C336fs | Likely pathogenic |
| CB160501 | BARD1 | NM_000465 | c.1073_1095AA | Splice_Site | Likely pathogenic |
| CB160515 | BARD1 | NM_000465 | c.1073_1095TA | Splice_Site | Likely pathogenic |
| CB161005 | BARD1 | NM_000465 | c.1046_1092GCAAACGGTGCCCTCAGAAAATATA | Splice_Site | Likely pathogenic |
| CB161736 | BARD1 | NM_000465 | c.1054delG | p.V352fs | Likely pathogenic |
| CB161958 | BARD1 | NM_000465 | c.1007dupG | p.C336fs | Likely pathogenic |
| CB170619 | BARD1 | NM_000465 | c.1142delG | p.S381fs | Likely pathogenic |
| CB170673 | BARD1 | NM_000465 | c.68_69insTCCGGGAACGAGCCTCGTTCCGC | p.A23fs | Pathogenic |
| CB171881 | BARD1 | NM_000465 | c.1007delG | p.C336fs | Likely pathogenic |
| CB171890 | BARD1 | NM_000465 | c.1008dupT | p.R337_T338delinsX | Likely pathogenic |
| CB180552 | BARD1 | NM_000465 | c.68_69insTCCGGGAACGAGCCTCGTTCCGC | p.A23fs | Pathogenic |
| CB180624 | BARD1 | NM_000465 | c.1021_1092GAGCACCAGTGGAGATTTTGTTAAGCAAACGGTGCCCTCAGAAAATATA | Splice_Site | Likely pathogenic |
| CB181393 | BARD1 | NM_000465 | c.1072_1094TC | Splice_Site | Likely pathogenic |
| CB181756 | BARD1 | NM_000465 | c.993_1092ATTTCTAAGAGATGTAGAACCAGCATTCTGAGCACCAGTGGAGATTTTGTTAAGCAAACGGTGCCCTCAGAAAATATA | Splice_Site | Likely pathogenic |
| CB181924 | BARD1 | NM_000465 | c.1007delG | p.C336fs | Likely pathogenic |
| CB181998 | BARD1 | NM_000465 | c.1072_1094AC | Splice_Site | Likely pathogenic |
| CB182682 | BARD1 | NM_000465 | c.1068_1092ATATA | Splice_Site | Likely pathogenic |
| CB182740 | BARD1 | NM_000465 | c.1067_1092TATA | Splice_Site | Likely pathogenic |
| CB200582 | BARD1 | NM_000465 | c.1067_1092TATA | Splice_Site | Likely pathogenic |
| CB170387 | BLM | NM_000057 | c.2907_2908del | p.S969fs | Likely pathogenic |
| CB170854 | BLM | NM_000057 | c.1144delT | p.L382X | Likely pathogenic |
| CB170967 | BLM | NM_000057 | c.1189delG | p.G397fs | Likely pathogenic |
| CB180086 | BLM | NM_000057 | c.1130delA | p.E377fs | Likely pathogenic |
| CB181208 | BLM | NM_000057 | c.897dupT | p.D299fs | Likely pathogenic |
| CB161687 | BRCA1 | NM_007297 | c.T1150G | p.L384V | Likely pathogenic |
| CB161719 | BRCA1 | NM_007297 | c.G5356T | p.V1786L | Likely pathogenic |
| CB170394 | BRCA1 | NM_007297 | c.A4633G | p.N1545D | Likely pathogenic |
| CB150962 | BRCA1 | NM_007297 | c.290delA | p.N97fs | Pathogenic |
| CB151019 | BRCA1 | NM_007297 | c.279delT | p.S93fs | Likely pathogenic |
| CB161297 | BRCA1 | NM_007297 | c.5329_5336del | p.I1777fs | Pathogenic |
| CB161377 | BRCA1 | NM_007297 | c.1969_1970del | p.N657fs | Pathogenic |
| CB170922 | BRCA1 | NM_007297 | c.5329_5336del | p.I1777fs | Pathogenic |
| CB171128 | BRCA1 | NM_007297 | c.266_300TAATTCTACAGAGTGAACCCGAAAATCATTCCTTG | Splice_Site | Likely pathogenic |
| CB171912 | BRCA1 | NM_007297 | c.3924_3927del | p.N1308fs | Pathogenic |
| CB172024 | BRCA1 | NM_007297 | c.285delC | p.P95fs | Likely pathogenic |
| CB193042 | BRCA1 | NM_007297 | c.3186_3188del | p.1062_1063del | Pathogenic |
| CB150741 | BRCA2 | NM_000059 | c.A9275G | p.Y3092C | Likely pathogenic |
| CB150854 | BRCA2 | NM_000059 | c.C3883T | p.Q1295X | Pathogenic |
| CB150883 | BRCA2 | NM_000059 | c.G7522A | p.G2508S | Likely pathogenic |
| CB151344 | BRCA2 | NM_000059 | c.C9599G | p.S3200X | Pathogenic |
| CB160627 | BRCA2 | NM_000059 | c.G7522A | p.G2508S | Likely pathogenic |
| CB161186 | BRCA2 | NM_000059 | c.G7522A | p.G2508S | Likely pathogenic |
| CB161432 | BRCA2 | NM_000059 | c.A9104G | p.Y3035C | Likely pathogenic |
| CB161859 | BRCA2 | NM_000059 | c.G6547T | p.E2183X | Pathogenic |
| CB162051 | BRCA2 | NM_000059 | c.C6952T | p.R2318X | Pathogenic |
| CB170139 | BRCA2 | NM_000059 | c.C9116T | p.P3039L | Likely pathogenic |
| CB170629 | BRCA2 | NM_000059 | c.G4363T | p.E1455X | Pathogenic |
| CB170953 | BRCA2 | NM_000059 | c.A9275G | p.Y3092C | Likely pathogenic |
| CB170963 | BRCA2 | NM_000059 | c.C3109T | p.Q1037X | Pathogenic |
| CB171347 | BRCA2 | NM_000059 | c.G7522A | p.G2508S | Likely pathogenic |
| CB171650 | BRCA2 | NM_000059 | c.G7522A | p.G2508S | Likely pathogenic |
| CB171958 | BRCA2 | NM_000059 | c.C274T | p.Q92X | Pathogenic |
| CB180465 | BRCA2 | NM_000059 | c.G8356A | p.A2786T | Likely pathogenic |
| CB180929 | BRCA2 | NM_000059 | c.G7522A | p.G2508S | Likely pathogenic |
| CB181299 | BRCA2 | NM_000059 | c.C9116T | p.P3039L | Likely pathogenic |
| CB181497 | BRCA2 | NM_000059 | c.C5682G | p.Y1894X | Pathogenic |
| CB181973 | BRCA2 | NM_000059 | c.G7522A | p.G2508S | Likely pathogenic |
| CB182054 | BRCA2 | NM_000059 | c.G7522A | p.G2508S | Likely pathogenic |
| CB191575 | BRCA2 | NM_000059 | c.G8356A | p.A2786T | Likely pathogenic |
| CB151494 | BRCA2 | NM_000059 | c.4408_4412del | p.I1470fs | Pathogenic |
| CB161424 | BRCA2 | NM_000059 | c.1886_1904del | p.L629fs | Likely pathogenic |
| CB171457 | BRCA2 | NM_000059 | c.5646delA | p.S1882fs | Likely pathogenic |
| CB171724 | BRCA2 | NM_000059 | c.7671_7672del | p.A2557fs | Pathogenic |
| CB171958 | BRCA2 | NM_000059 | c.272_316ATTGGTACAGGTATCTAATTCTCCTGTAAAAGAATTAGATAAATT | Splice_Site | Likely pathogenic |
| CB181779 | BRCA2 | NM_000059 | c.2176delG | p.V726fs | Pathogenic |
| CB191422 | BRCA2 | NM_000059 | c.4795_4797del | p.1599_1599del | Pathogenic |
| CB140314 | BRIP1 | NM_032043 | c.C1255T | p.R419W | Likely pathogenic |
| CB150194 | BRIP1 | NM_032043 | c.918+1G>A | Splice_Site | Likely pathogenic |
| CB160969 | BRIP1 | NM_032043 | c.C1741T | p.R581X | Pathogenic |
| CB170527 | BRIP1 | NM_032043 | c.G2287A | p.G763S | Likely pathogenic |
| CB180031 | BRIP1 | NM_032043 | c.C1255T | p.R419W | Likely pathogenic |
| CB201105 | BRIP1 | NM_032043 | c.C1315T | p.R439X | Pathogenic |
| CB160442 | BRIP1 | NM_032043 | c.2464dupT | p.Y822fs | Pathogenic |
| CB170306 | BRIP1 | NM_032043 | c.1197_1199del | p.399_400del | Pathogenic |
| CB170854 | BRIP1 | NM_032043 | c.1928_1935TTCACAGG | Splice_Site | Likely pathogenic |
| CB171165 | BRIP1 | NM_032043 | c.2992_2993del | p.K998fs | Likely pathogenic |
| CB171757 | BRIP1 | NM_032043 | c.2947dupA | p.I983fs | Likely pathogenic |
| CB180108 | BRIP1 | NM_032043 | c.2464dupT | p.Y822fs | Pathogenic |
| CB171407 | CDKN2A | NM_058197 | c.254delA | p.K85fs | Likely pathogenic |
| CB151600 | CHEK2 | NM_007194 | c.1375+1G>A | Splice_Site | Likely pathogenic |
| CB160407 | CHEK2 | NM_007194 | c.909-1G>A | Splice_Site | Likely pathogenic |
| CB160701 | CHEK2 | NM_007194 | c.1260-1G>A | Splice_Site | Likely pathogenic |
| CB161504 | CHEK2 | NM_007194 | c.G643T | p.A215S | Likely pathogenic |
| CB161746 | CHEK2 | NM_007194 | c.C232T | p.Q78X | Pathogenic |
| CB171881 | CHEK2 | NM_007194 | c.847-2A>G | Splice_Site | Likely pathogenic |
| CB171962 | CHEK2 | NM_007194 | c.G917C | p.G306A | Likely pathogenic |
| CB172199 | CHEK2 | NM_007194 | c.246_260del | p.82_87del | Pathogenic |
| CB180783 | CHEK2 | NM_007194 | c.1357dupG | p.A453fs | Likely pathogenic |
| CB192886 | CHEK2 | NM_007194 | c.343delC | p.Q115fs | Likely pathogenic |
| CB170043 | EPCAM | NM_002354 | c.491+1G>A | Splice_Site | Pathogenic |
| CB182253 | EPCAM | NM_002354 | c.T753G | p.Y251X | Likely pathogenic |
| CB150522 | EPCAM | NM_002354 | c.741dupT | p.T247fs | Likely pathogenic |
| CB141551 | MLH1 | NM_000249 | c.T1916G | p.L639X | Pathogenic |
| CB141659 | MLH1 | NM_000249 | c.G194A | p.G65D | Likely pathogenic |
| CB141813 | MLH1 | NM_000249 | c.G299C | p.R100P | Pathogenic |
| CB150044 | MLH1 | NM_000249 | c.T849G | p.Y283X | Likely pathogenic |
| CB150095 | MLH1 | NM_000249 | c.G199A | p.G67R | Pathogenic |
| CB150112 | MLH1 | NM_000249 | c.A1709G | p.N570S | Likely pathogenic |
| CB150115 | MLH1 | NM_000249 | c.A644C | p.N215T | Likely pathogenic |
| CB150272 | MLH1 | NM_000249 | c.G1731A | p.S577S | Pathogenic |
| CB150456 | MLH1 | NM_000249 | c.G199A | p.G67R | Pathogenic |
| CB150821 | MLH1 | NM_000249 | c.C114G | p.N38K | Pathogenic |
| CB151397 | MLH1 | NM_000249 | c.C2093G | p.S698X | Pathogenic |
| CB151529 | MLH1 | NM_000249 | c.678-2A>G | Splice_Site | Likely pathogenic |
| CB151668 | MLH1 | NM_000249 | c.C632G | p.S211X | Pathogenic |
| CB160584 | MLH1 | NM_000249 | c.A883G | p.S295G | Pathogenic |
| CB160873 | MLH1 | NM_000249 | c.453+1G>A | Splice_Site | Likely pathogenic |
| CB161027 | MLH1 | NM_000249 | c.A644C | p.N215T | Likely pathogenic |
| CB161161 | MLH1 | NM_000249 | c.G323C | p.S108T | Likely pathogenic |
| CB161199 | MLH1 | NM_000249 | c.G199A | p.G67R | Pathogenic |
| CB161334 | MLH1 | NM_000249 | c.C114G | p.N38K | Pathogenic |
| CB161504 | MLH1 | NM_000249 | c.G1154A | p.R385H | Likely pathogenic |
| CB161547 | MLH1 | NM_000249 | c.A191G | p.N64S | Likely pathogenic |
| CB161576 | MLH1 | NM_000249 | c.C2059T | p.R687W | Pathogenic |
| CB161685 | MLH1 | NM_000249 | c.C114G | p.N38K | Pathogenic |
| CB161687 | MLH1 | NM_000249 | c.A1136C | p.Y379S | Likely pathogenic |
| CB161758 | MLH1 | NM_000249 | c.G1154A | p.R385H | Likely pathogenic |
| CB161990 | MLH1 | NM_000249 | c.C1730T | p.S577L | Likely pathogenic |
| CB162100 | MLH1 | NM_000249 | c.G199A | p.G67R | Pathogenic |
| CB170306 | MLH1 | NM_000249 | c.G1717T | p.V573F | Likely pathogenic |
| CB171143 | MLH1 | NM_000249 | c.1990-2A>C | Splice_Site | Likely pathogenic |
| CB171344 | MLH1 | NM_000249 | c.A1984G | p.T662A | Likely pathogenic |
| CB171501 | MLH1 | NM_000249 | c.G100A | p.E34K | Likely pathogenic |
| CB171713 | MLH1 | NM_000249 | c.A1136C | p.Y379S | Likely pathogenic |
| CB171915 | MLH1 | NM_000249 | c.C1684T | p.Q562X | Pathogenic |
| CB172007 | MLH1 | NM_000249 | c.A1984G | p.T662A | Likely pathogenic |
| CB180289 | MLH1 | NM_000249 | c.A244G | p.T82A | Likely pathogenic |
| CB180297 | MLH1 | NM_000249 | c.C676T | p.R226X | Pathogenic |
| CB180402 | MLH1 | NM_000249 | c.G67T | p.E23X | Pathogenic |
| CB180446 | MLH1 | NM_000249 | c.G1731A | p.S577S | Pathogenic |
| CB180579 | MLH1 | NM_000249 | c.G2041A | p.A681T | Pathogenic |
| CB180635 | MLH1 | NM_000249 | c.C256T | p.Q86X | Pathogenic |
| CB181046 | MLH1 | NM_000249 | c.C256T | p.Q86X | Pathogenic |
| CB181198 | MLH1 | NM_000249 | c.G199A | p.G67R | Pathogenic |
| CB181278 | MLH1 | NM_000249 | c.T2248G | p.Y750D | Likely pathogenic |
| CB181467 | MLH1 | NM_000249 | c.453+1G>A | Splice_Site | Likely pathogenic |
| CB182374 | MLH1 | NM_000249 | c.C350T | p.T117M | Pathogenic |
| CB182556 | MLH1 | NM_000249 | c.C676T | p.R226X | Pathogenic |
| CB182741 | MLH1 | NM_000249 | c.C350T | p.T117M | Pathogenic |
| CB190071 | MLH1 | NM_000249 | c.C1630T | p.Q544X | Pathogenic |
| CB190483 | MLH1 | NM_000249 | c.C298T | p.R100X | Pathogenic |
| CB191530 | MLH1 | NM_000249 | c.453+1G>T | Splice_Site | Likely pathogenic |
| CB191546 | MLH1 | NM_000249 | c.C2093G | p.S698X | Pathogenic |
| CB191586 | MLH1 | NM_000249 | c.1039-1G>A | Splice_Site | Pathogenic |
| CB191665 | MLH1 | NM_000249 | c.C2093G | p.S698X | Pathogenic |
| CB192566 | MLH1 | NM_000249 | c.T849G | p.Y283X | Likely pathogenic |
| CB192981 | MLH1 | NM_000249 | c.790+1G>A | Splice_Site | Pathogenic |
| CB200812 | MLH1 | NM_000249 | c.C793A | p.R265S | Pathogenic |
| CB201221 | MLH1 | NM_000249 | c.1558+1G>A | Splice_Site | Likely pathogenic |
| CB131684 | MLH1 | NM_000249 | c.1062delC | p.G354fs | Likely pathogenic |
| CB140933 | MLH1 | NM_000249 | c.694dupG | p.I231fs | Likely pathogenic |
| CB150346 | MLH1 | NM_000249 | c.2071_2072insTCCGGAAGTATTCCATCCGGAAGCAG | p.I691fs | Likely pathogenic |
| CB151690 | MLH1 | NM_000249 | c.1845_1847del | p.615_616del | Pathogenic |
| CB151772 | MLH1 | NM_000249 | c.1484delC | p.T495fs | Pathogenic |
| CB161514 | MLH1 | NM_000249 | c.1484dupC | p.T495fs | Pathogenic |
| CB170726 | MLH1 | NM_000249 | c.1103delC | p.S368fs | Likely pathogenic |
| CB170886 | MLH1 | NM_000249 | c.1484dupC | p.T495fs | Pathogenic |
| CB180283 | MLH1 | NM_000249 | c.2156dupT | p.I719fs | Pathogenic |
| CB180702 | MLH1 | NM_000249 | c.1062delC | p.G354fs | Likely pathogenic |
| CB181192 | MLH1 | NM_000249 | c.1484delC | p.T495fs | Pathogenic |
| CB181296 | MLH1 | NM_000249 | c.1100_1101del | p.T367fs | Likely pathogenic |
| CB181387 | MLH1 | NM_000249 | c.1100dupC | p.T367fs | Likely pathogenic |
| CB182253 | MLH1 | NM_000249 | c.1667delG | p.S556fs | Likely pathogenic |
| CB192866 | MLH1 | NM_000249 | c.599dupC | p.T200fs | Likely pathogenic |
| CB193042 | MLH1 | NM_000249 | c.756dupA | p.S252fs | Pathogenic |
| CB200289 | MLH1 | NM_000249 | c.1484dupC | p.T495fs | Pathogenic |

**Supplementary Table 3. Susceptibility of respective mutant gene to colorectal cancer (n=8270).**

| Gene | This study  n (%) | Chinese control*  n (%) | p value | OR | 95%CI |
| --- | --- | --- | --- | --- | --- |
| *MLH1* | 74 (0.89) | 1 (0.0053)^#^ | < 0.001 | 168.527 | 29.34-6473.80 |
| *MSH2* | 63 (0.76) | 6 (0.0318)^#^ | < 0.001 | 23.920 | 10.39-67.80 |
| *MSH6* | 49 (0.59) | 10 (0.0531)^#^ | < 0.001 | 11.160 | 5.58-24.72 |
| *ATM* | 29 (0.35) | 12 (0.0567) | 0.001 | 3.094 | 1.53-6.66 |
| *PMS2* | 28 (0.34) | 16 (0.0849)^#^ | < 0.001 | 3.987 | 2.08-7.89 |
| *APC* | 16 (0.19) | 1 (0.0047) | < 0.001 | 20.484 | 3.18-855.70 |
| *POLE* | 17 (0.21) | 7 (0.0331) | 0.012 | 3.109 | 1.2-8.87 |
| *TP53* | 9 (0.11) | 2 (0.0094) | 0.014 | 5.760 | 1.19-54.76 |
| *BARD1* | 28 (0.34) | 13 (0.0614) | 0.002 | 2.757 | 1.38-5.80 |
| *RAD50* | 17(0.21) | 7 (0.0331) | 0.012 | 3.109 | 1.23-8.87 |

^*^allele frequency

^#^ Data adopted from this study^36^

**Supplementary Table 4. Demographic and clinical characteristics of 2181 high-risk hereditary CRC patients’ variant status.**

| Variables | Total population  N = 2181 | Germline mutation carriers | | | | |
| --- | --- | --- | --- | --- | --- | --- |
|  |  | MMR genes | HR genes | APC | Other | None |
|  |  | N = 199 | N = 141 | N = 16 | N = 65 | N = 1760 |
| Age (years) | 50.8 ± 12.8 (17-94) | 48.6 ±12.3 (18-81) | 51.2 ± 12.8 (22-82) | 40.6 ± 10.7 (17-56) | 50.8 ± 13.3 (26-86) | 51.1 ± 12.7 (19-94) |
| < 50 | 1298 (59.5) | 120 (60.3) | 83 (58.9) | 13 (81.3) | 42 (64.6) | 1040 (59.1) |
| ≥ 50 | 883 (40.5) | 79 (39.7) | 58 (41.1) | 3 (18.7) | 23 (35.4) | 720 (40.9) |
| Gender |  |  |  |  |  |  |
| Male | 1215 (55.7) | 113 (56.8) | 81 (57.4) | 9 (56.2) | 36 (55.4) | 976 (55.5) |
| Female | 966 (44.3) | 86 (43.2) | 60 (42.6) | 7 (43.8) | 29 (44.6) | 784 (44.5) |
| Chronic disease |  |  |  |  |  |  |
| Presence | 172 (7.9) | 12 (6.0) | 11 (7.8) | 0 (0) | 5 (7.7) | 144 (8.2) |
| Absence | 2009 (92.1) | 187 (94.0) | 130 (92.2) | 16 (100.0) | 60 (92.3) | 1616 (91.8) |
| CEA (ng/ml) |  |  |  |  |  |  |
| ≥ 5.2 | 878 (40.3) | 62 (31.2) | 67 (47.5) | 5 (31.2) | 28 (43.1) | 716 (40.7) |
| < 5.2 | 1303 (59.7) | 137 (68.8) | 74 (52.5) | 11 (68.8) | 37 (56.9) | 1044 (59.3) |
| CRC site |  |  |  |  |  |  |
| Right colon | 490 (22.5) | 71 (35.7) | 24 (17.0) | 2 (12.5) | 11 (16.9) | 382 (21.7) |
| Transverse colon | 144 (6.6) | 16 (8.0) | 10 (7.1) | 3 (18.7) | 5 (7.7) | 112 (6.4) |
| Left colon | 599 (27.5) | 47 (23.6) | 49 (34.8) | 1 (6.3) | 19 (29.2) | 481 (27.3) |
| Rectum | 864 (39.6) | 45 (22.6) | 56 (39.7) | 6 (37.5) | 27 (41.5) | 730 (41.5) |
| Multiple | 84 (3.8) | 20 (10.1) | 2 (1.4) | 4 (25.0) | 3 (4.6) | 55 (3.1) |
| Tumor size ^a^(cm) | 4.2 ± 2.1 (0.2-16.8) | 5.0 ± 2.4 (0.5-13.0) | 3.9 ± 1.9 (0.4-11.0) | 3.9 ± 1.5 (2.0-7.0) | 4.1 ± 2.0 (1.2-12) | 4.1 ± 2.1 (0.2-16.8) |
| Pathological type |  |  |  |  |  |  |
| Adenocarcinoma | 1850 (84.8) | 174 (87.4) | 113 (80.1) | 12 (75.0) | 57 (87.7) | 1494 (84.9) |
| Mucinous | 254 (11.6) | 20 (10.1) | 21 (14.9) | 3 (18.7) | 7 (10.8) | 203 (11.5) |
| Signer ring cell carcinoma | 77 (3.6) | 5 (2.5) | 7 (5.0) | 1 (6.3) | 1 (1.5) | 63 (3.6) |
| Differentiation grade |  |  |  |  |  |  |
| Well | 112 (5.1) | 7 (3.5) | 8 (5.7) | 1 (6.3) | 5 (7.7) | 91 (5.2) |
| Moderately | 1394 (63.9) | 116 (58.3) | 91 (64.5) | 10 (62.5) | 48 (73.8) | 1129 (64.1) |
| Poorly | 675 (31.0) | 76 (38.2) | 42 (29.8) | 5 (31.2) | 12 (18.5) | 540 (30.7) |
| Cancerous node |  |  |  |  |  |  |
| Occurrence | 322 (14.8) | 15 (7.5) | 26 (18.4) | 4 (25.0) | 19 (29.2) | 264 (15.0) |
| Absence | 1859 (85.2) | 184 (92.5) | 115 (81.6) | 7 (75.0) | 46 (70.8) | 1496 (85.0) |
| Vascular invasion |  |  |  |  |  |  |
| Occurrence | 597 (27.4) | 41 (20.6) | 39 (27.7) | 5 (31.2) | 17 (26.2) | 495 (28.1) |
| Absence | 1584 (72.6) | 158 (79.4) | 102 (72.3) | 11 (68.8) | 48 (73.8) | 1265 (71.9) |
| Perineural invasion |  |  |  |  |  |  |
| Occurrence | 617 (28.3) | 42 (21.1) | 43 (30.5) | 5 (31.2) | 19 (29.2) | 508 (28.9) |
| Absence | 1564 (71.7) | 157 (78.9) | 98 (69.5) | 11 (68.8) | 46 (70.8) | 1252 (71.1) |
| TNM stage |  |  |  |  |  |  |
| 0 | 57 (2.6) | 3 (1.5) | 4 (2.8) | 1 (6.3) | 2 (3.1) | 47 (2.7) |
| I | 409 (18.8) | 36 (18.1) | 28 (19.9) | 4 (25.0) | 13 (20.0) | 328 (18.6) |
| II | 711 (32.6) | 91 (45.8) | 42 (29.8) | 3 (18.7) | 22 (33.8) | 553 (31.4) |
| III | 770 (35.3) | 56 (28.1) | 54 (38.3) | 6 (37.5) | 21 (32.3) | 633 (36.0) |
| IV | 234 (10.7) | 13 (6.5) | 13 (9.2) | 2 (12.5) | 7 (10.8) | 199 (11.3) |
| IHC MMR |  |  |  |  |  |  |
| Deficiency | 401 (18.4) | 180 (90.5) | 20 (14.2) | 2 (12.5) | 11 (16.9) | 188 (10.7) |
| Proficiency | 1394 (63.9) | 0 (0) | 89 (63.1) | 7 (43.8) | 41 (63.1) | 1257 (71.4) |
| Not applicable | 386 (17.7) | 19 ( 9.5) | 32 (22.7) | 7 (43.8) | 13 (20.0) | 315 (17.9) |
| *RAS* genotype |  |  |  |  |  |  |
| Wild type | 478 (21.9) | 63 (31.7) | 25 (17.7) | 4 (25.0) | 11 (16.9) | 375 (21.3) |
| Mutant type | 402 (18.4) | 43 (21.6) | 26 (18.5) | 4 (25.0) | 9 (13.8) | 320 (18.2) |
| Not applicable | 1301 (59.7) | 93 (46.7) | 90 (64.8) | 8 (50.0) | 45 (68.3) | 1065 (60.5) |
| *BRAF* genotype |  |  |  |  |  |  |
| Wild type | 817 (37.5) | 102 (51.3) | 50 (35.5) | 7 (43.8) | 17 (26.2) | 641 (36.4) |
| Mutant type | 63(2.9) | 4 (2.0) | 1 (0.7) | 1 (6.3) | 3 (4.5) | 54 (3.1) |
| Not applicable | 1301 (59.6) | 93 (46.7) | 90 (64.8) | 8 (50.0) | 45 (68.3) | 1065 (60.5) |
| Microsatellite |  |  |  |  |  |  |
| Stable | 77 (3.5) | 4 (2.0) | NA | 1 (6.3) | NA | 67 (3.8) |
| Instability-low | 4 (0.2) | 2 (1.0) | NA | NA | NA | 2 (0.1) |
| Instability-high | 82 (3.8) | 31 (15.6) | NA | NA | NA | 49 (2.8) |
| Not applicable | 2018 (92.5) | 162 (81.4) | 141 (100%) | 15 (93.7) | 141 (100%) | 1642 (93.3) |

**Supplementary Table 5. Cancer history of 2181 high-risk hereditary CRC patients.**

| Variables | Total population  N = 2181 | Germline mutation carriers | | | | |
| --- | --- | --- | --- | --- | --- | --- |
|  |  | MMR genes | HR genes | APC | Other | None |
|  |  | N = 199 | N = 141 | N = 16 | N = 65 | N = 1760 |
| Self-reported multiple primary colorectal cancer | 187 (8.6) | 48 (24.1) | 12 (8.5) | 5 (31.3) | 5 (7.7) | 117 (6.6) |
| Synchronous CRC | 122 (5.6) | 26 (13.1) | 6 (4.3) | 4 (25.0) | 3 (4.6) | 83 (4.7) |
| Metachronous CRC | 81 (3.7) | 28 (14.4) | 6 (4.3) | 1 (5.0) | 4 (6.2) | 42 (2.4) |
| Self–reported extracolonic cancer | 250 (11.5) | 31 (15.6) | 25 (17.7) | 3 (18.8) | 7 (10.8) | 184 (10.5) |
| Gastrointestinal cancer | 37 (1.7) | 11 (5.5) | 2 (1.4) | NA | NA | 24 (1.4) |
| Gastric cancer | 27 (1.2) | 6 (3.0) | 2(1.4) |  |  | 19 (1.1) |
| Small bowel cancer | 9 (0.4) | 4 (2.0) | NA |  |  | 5 (0.3) |
| Other | 1 (0.05) | 1 (0.5) | NA |  |  | NA |
| Gynecological cancer | 107 (11.1)^a^ | 16 (18.6) ^a^ | 12 (20) ^a^ | NA | 2 (6.9) ^a^ | 77 (9.8) ^a^ |
| Uterine cancer | 32 (3.3) ^a^ | 13 (15.3) ^a^ | 2 (3.3) ^a^ |  | 1 (3.4) ^a^ | 24 (3.1) ^a^ |
| ovarian cancer | 21 (2.2) ^a^ | 1 (1.2) ^a^ | 5 (8.3) ^a^ |  | NA | 15 (1.9) ^a^ |
| Breast cancer | 47 (4.9) ^a^ | 3 (3.5) ^a^ | 5 (8.3) ^a^ |  | 1 (3.4) ^a^ | 38 (4.8) ^a^ |
| Urogenital cancer | 50 (2.3) | 3 (1.5) | 7 (5.0) | NA | 1 (1.5) | 39 (2.2) |
| Bladder cancer | 18 (0.8) | 1 (0.5) | 2 (1.4) |  | NA | 15 (0.9) |
| Renal cancer | 17 (0.8) | 1 (0.5) | 4 (2.8) |  | NA | 12 (0.7) |
| Prostate cancer | 15 (7.5) | 1 (0.5) | 1 (0.7) |  | 1 (1.5) | 12 (0.7) |
| Hepatobiliary cancer | 10 (0.5) | 1 (0.5) | NA | 1 (6.3) | NA | 8 (0.5) |
| Pancreatic cancer | 4 (0.2) | NA | 1 (0.7) | NA | NA | 3 (0.2) |
| Other cancer | 65 (3.0) | 6 (3.0) | 6 (4.3) | 2 (12.5) | 5 (7.7) | 46 (2.6) |

^a.^analyzed in female patients

**Supplementary Table 6. Univariate and multivariate analyses of prognostic factors for recurrence free survival.**

| Variable | Univariate analysis | |  | Multivariate analysis | | |
| --- | --- | --- | --- | --- | --- | --- |
|  | χ^2^ value | *p* value |  | Odds ratio | 95% CI | *p* value |
| Sex: male vs. female | 0.962 | 0.327 |  |  |  |  |
| Chronic: presence vs. absence | 1.323 | 0.250 |  |  |  |  |
| Onset age: early vs. late | 19.146 | < 0.001 |  | 1.037 | 0.870-1.234 | 0.687 |
| Family cancer history: presence vs. absence | 1.387 | 0.239 |  |  |  |  |
| Family CRC history: presence vs. absence | 1.625 | 0.202 |  |  |  |  |
| CEA: < vs.> 5μg/L | 67.500 | < 0.001 |  | 1.530 | 1.299-1.103 | 0.002 |
| CRC site: right colon vs. transverse colon vs. left colon vs. rectum vs. multiple | 5.235 | 0.264 |  |  |  |  |
| Pathological type: adenocarcinoma vs. mucinous vs. signer ring cell carcinoma | 31.733 | < 0.001 |  | 1.011 | 0.860-1.189 | 0.891 |
| Differentiation grade: well vs. moderate vs. poor | 69.142 | < 0.001 |  | 1.346 | 1.129-1.604 | 0.001 |
| Vascular invasion: occurrence vs. absence | 212.324 | < 0.001 |  | 1.264 | 1.056-1.512 | 0.011 |
| Perineural invasion: occurrence vs. absence | 177.979 | < 0.001 |  | 1.283 | 1.076-1.531 | 0.006 |
| Cancerous nodule: occurrence vs. absence | 268.019 | < 0.001 |  | 1.359 | 1.129-1.637 | 0.001 |
| TNM stage: I vs. II vs. III vs. IV | 920.925 | <0.001 |  | 2.652 | 2.352-2.991 | < 0.001 |
| Metachronous primary CRC: occurrence vs. absence | 9.255 | 0.002 |  | 0.482 | 0.273-0.851 | 0.012 |
| Primary extracolonic cancer: occurrence vs. absence | 0.216 | 0.642 |  |  |  |  |
| MMR by IHC: pMMR vs. dMMR^a^ | 50.147 | <0.001 |  | 0.631 | 0.470-0.849 | 0.002 |
| *KRAS* genotype: wild vs. mutant^b^ | 1.362 | 0.243 |  |  |  |  |
| *BRAF V600E*: presence vs. absence^c^ | 0.027 | 0.869 |  |  |  |  |
| Germline mutation: presence vs. absence | 9.737 | 0.002 |  |  |  |  |
| Germline mutation: MMR vs. HR vs. APC vs. other vs. none | 23.055 | <0.001 |  | 1.008 | 0.909-1.118 | 0.879 |

^a,b,c^. analyzed in patients with result.

**Supplementary Table 7. Univariate and multivariate analyses of prognostic factors for overall survival.**

| Variable | Univariate analysis | |  | Multivariate analysis | | |
| --- | --- | --- | --- | --- | --- | --- |
|  | χ^2^ value | *p* value |  | Odds ratio | 95% CI | *p* value |
| Sex: male vs. female | 0.080 | 0.777 |  |  |  |  |
| Chronic: presence vs. absence | 1.669 | 0.196 |  |  |  |  |
| Onset age: early vs. late | 7.838 | 0.005 |  | 0.892 | 0.725-1.098 | 0.280 |
| Family cancer history: presence vs. absence | 0.137 | 0.711 |  |  |  |  |
| Family CRC history: presence vs. absence | 0.332 | 0.565 |  |  |  |  |
| CEA: < vs.> 5μg/L | 48.639 | <0.001 |  | 1.241 | 1.019-1.512 | 0.032 |
| CRC site: right colon vs. transverse colon vs. left colon vs. rectum vs. multiple | 4.989 | 0.288 |  |  |  |  |
| Pathological type: adenocarcinoma vs. mucinous vs. signer ring cell carcinoma | 57.544 | <0.001 |  | 1.339 | 1.124-1.596 | 0.001 |
| Differentiation grade: well vs. moderate vs. poor | 73.833 | <0.001 |  | 1.326 | 1.071-1.642 | 0.010 |
| Vascular invasion: occurrence vs. absence | 170.092 | <0.001 |  | 1.265 | 1.017-1.573 | 0.035 |
| Perineural invasion: occurrence vs. absence | 144.139 | <0.001 |  | 1.405 | 1.137-1.736 | 0.002 |
| Cancerous nodule: occurrence vs. absence | 231.313 | <0.001 |  | 1.417 | 1.143-1.756 | 0.001 |
| TNM stage: I vs. II vs. III vs. IV | 759.888 | <0.001 |  | 3.197 | 2.755-3.709 | <0.001 |
| Metachronous primary CRC: occurrence vs. absence | 7.767 | 0.005 |  | 0.456 | 0.256-0.811 | 0.008 |
| Primary extracolonic cancer: occurrence vs. absence | 0.205 | 0.650 |  |  |  |  |
| MMR by IHC: pMMR vs. dMMR^a^ | 32.311 | <0.001 |  | 0.632 | 0.436-0.915 | 0.015 |
| *KRAS* genotype: wild vs. mutant^b^ | 1.482 | 0.224 |  |  |  |  |
| *BRAF V600E*: presence vs. absence^c^ | 1.439 | 0.230 |  |  |  |  |
| Germline mutation: presence vs. absence | 5.389 | 0.020 |  |  |  |  |
| Germline mutation: MMR vs. HR vs. APC vs. other vs. none | 19.946 | 0.001 |  | 1.058 | 0.936-1.195 | 0.368 |

^a,b,c^. analyzed in patients with result.
