# Supplementary figures and images for: The Largest Chinese Cohort Study Indicates Homologous Recombination Pathway Gene Mutations as Another Major Genetic Risk Factor for Colorectal Cancer with Heterogeneous Clinical Phenotypes
Source: Research (Wash D C). 2023 Oct 17;6:0249. doi: 10.34133/research.0249 (PMC10581333; doi:10.34133/research.0249)

# Supplementary Figure. 1

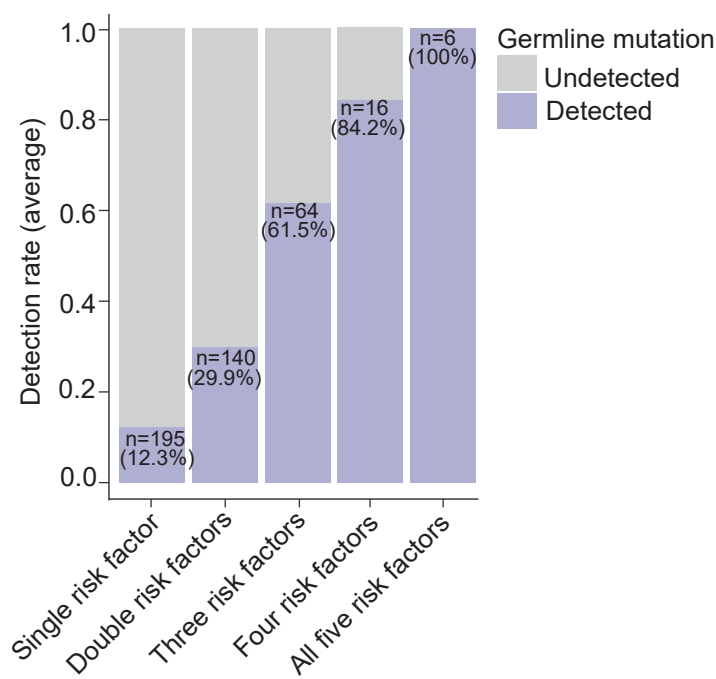

# Supplementary Figure. 2

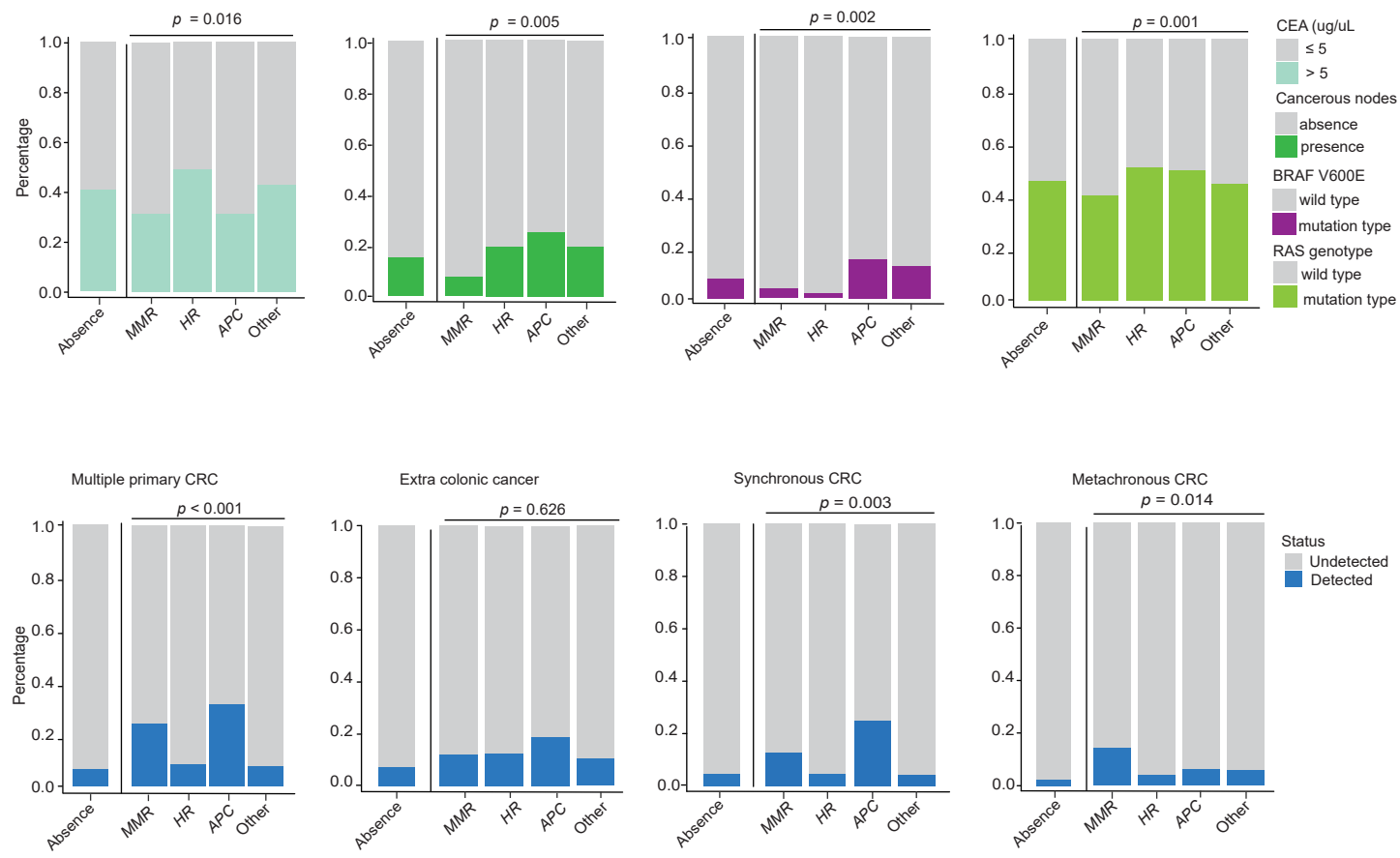

# Supplementary Figure. 3

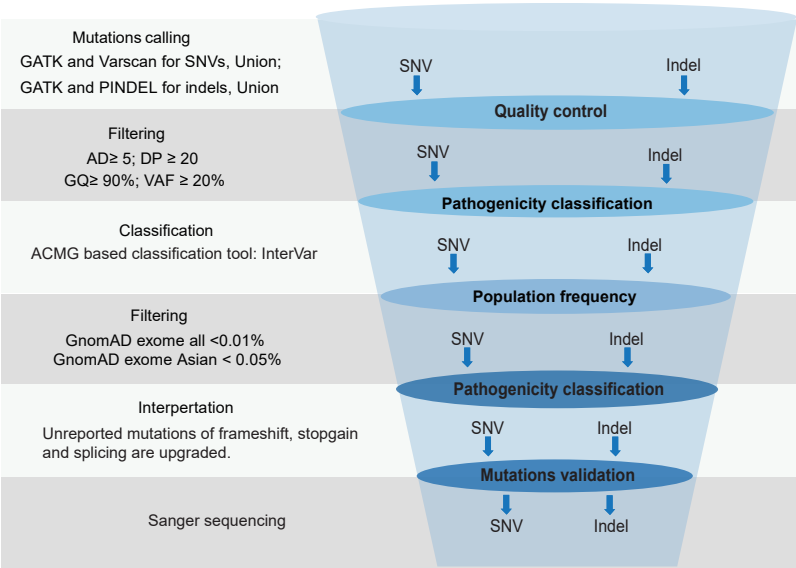

Supplement: Supplementary 1 — Figs. S1 to S3 Tables S1 to S7 [file research.0249.f1.zip › supplementary figures.pdf]
